# Revealing gut microbiota profiles and their influencing factors in commercial boars of three breeds by a large-scale metagenome study

**DOI:** 10.3389/fmicb.2026.1825304

**Published:** 2026-05-20

**Authors:** Zikang Hu, Congying Chen

**Affiliations:** National Key Laboratory of Pig Genetic Improvement and Germplasm Innovation, Jiangxi Agricultural University, Nanchang, China

**Keywords:** age, boar, breed, gut microbiota, multivariable models, rearing environment, shotgun metagenomic sequencing

## Abstract

Boars play a critical role in pig production. Numerous studies have reported important effects of the gut microbiota on pig production traits. However, whether the gut microbiota is associated with reproduction traits in boars remains largely unknown. Understanding the gut microbial composition and its influencing factors in large-scale boar populations is an essential first step to investigate this association. In this study, shotgun metagenomic sequencing was performed on fecal samples of 1,651 commercial boars from three breeds raised in three pig farms to uncover their gut microbial structures. We observed significant differences in boar gut microbial compositions across three breeds, even when raised in the same farm. Permutational multivariate analysis of variance (PERMANOVA) within-farm breeds and with-age stages found that the effect size of each factor on boar gut microbial composition varied across farms and age stages. Breeds accounted for 2% ~ 9% of the variance of boar gut microbial compositions in different farms. We then identified gut microbial taxa enriched in each boar breed using MaAsLin2. Lactic acid and butyrate-producing taxa, such as *Lactobacillus amylovorus* and *Faecalibacterium prausnitzii*, were enriched in Duroc boars; *Akkermansia muciniphila* and *Lactobacillus reuteri* showed the enrichment in Landrace boars, accompanied by increased relative abundance of Enterobacteriaceae members. Meanwhile, the species from *Bacteroides*, *Prevotella*, and *Treponema* had higher abundances in the gut of Large White pigs than in the other two pig breeds. We also identified bacterial species enriched in each of the three age stages. These breed and age-associated microbial enrichment patterns might reflect the combined effects of long-term genetic selection of pig breeds, age, and differences in feeding diets. The results of this study provide important insights for further investigating the effects of gut microbiota on boar reproductive traits and for developing strategies to modulate the gut microbiota to improve boar health and production performance.

## 1 Introduction

Microbial communities that colonize the mammalian gastrointestinal tract exhibit extensive diversity (Brooks et al., 2016; Youngblut et al., 2018; Liufu et al., 2024). Their compositional and functional profiles undergo dynamic succession during distinct stages of host development and across the lifespan (Stewart et al., 2018). This complex ecosystem is widely regarded as a microbial organ of the host, playing a central role in maintaining host physiological homeostasis and contributing to the development of diverse diseases (Bäckhed et al., 2005). The gut microbiota is extensively involved in a wide range of physiological processes, including nutrient digestion and absorption, regulation of energy metabolism, maintenance of immune homeostasis, and modulation of neurobehavioral functions. Through the fermentation and biotransformation of dietary substrates, it generates bioactive metabolites, such as short-chain fatty acids (SCFAs), which participate in energy homeostasis and the regulation of key metabolic pathways. Mutualistic interactions at the community level help maintain ecological stability and functional redundancy within the microbial ecosystem. In this symbiotic relationship, the host provides a stable anaerobic environment and complex polysaccharides, which are metabolized by microorganisms into absorbable carbon sources and energy substrates. This reciprocal metabolic exchange forms a dynamic and tightly integrated host–microbiota interaction (Sekirov et al., 2010).

Pigs represent an important livestock species and have also been widely used as a valuable biomedical model (Huang and Chen, 2023). The porcine gastrointestinal tract harbors a diverse and complex microbial community, whose composition and functional capacity are shaped by host genetics, age, diets, and environmental exposures (Lu et al., 2013). Previous studies have demonstrated that host genetic variation was associated with the taxonomic composition and functional potential of the gut microbiota (Zhang et al., 2015). Landrace, Large White, and Duroc pigs are major commercial breeds that exhibit marked differences in growth performance, reproductive traits, and digestive capacity, reflecting their distinct genetic backgrounds (Bergamaschi et al., 2020; Yu et al., 2020). These genetic differences may contribute to breed-specific gut microbiota structure (Yang et al., 2014). In large-scale pig production systems, boars serve as the primary breeding stock at the apex of the breeding pyramid, and their genetic characteristics directly influence the productive and economic performance of commercial pig herds. Boars at different developmental stages display marked variation in reproductive endocrine activity and metabolic status. During the juvenile period, rapid gonadal development occurs. In response to gonadotropin stimulation, testicular testosterone secretion increases, accompanied by the onset and acceleration of spermatogenesis as well as endocrine maturation (Ramaswamy and Weinbauer, 2014; Spaziani et al., 2020; Lei et al., 2025). Concurrently, systemic energy demands increase substantially to support reproductive development (Allrich et al., 1983). During sexual maturity, testicular development and endocrine function reach a relatively stable state (Almeida et al., 2006; Bhattacharya et al., 2023). In contrast, the aged stage shows reduced circulating testosterone levels, declining semen quality, and compromised antioxidant and immune functions (Aitken, 2023; Walke et al., 2023; Sá et al., 2025). These changes may increase susceptibility to metabolic imbalance and environmental stressors (Tsakmakidis et al., 2012; Hu et al., 2024). The gut microbiota undergoes stage-specific alterations in composition and function during host aging and responds to energy metabolism and endocrine regulation (O'Toole and Claesson, 2010; O’Toole and Jeffery, 2015; Ghosh et al., 2022). Therefore, physiological differences observed among boars at distinct developmental stages may be associated with variation in their gut microbial profiles. However, previous studies have primarily focused on sows or castrated boars, and systematic comparisons of fecal microbial community structure in intact breeding boars remain scarce. In addition, most studies employed 16S ribosomal RNA gene (16S rRNA) sequencing, which offers limited taxonomic resolution. This methodological limitation restricts the characterization of microbial composition and genomic features, thereby hindering a more detailed analysis of community structural differences.

To investigate the association of gut microbiota with boar reproduction traits in the future, this study systematically analyzed the compositional profiles of the gut microbiota in boars and investigated the factors influencing the gut microbial community variation based on metagenomic sequencing data of 1,651 fecal samples from three commercial boar breeds, namely Duroc, Landrace, and Large White. Significant differences in gut microbial composition were observed across breeds and rearing environments. Multifactorial analyses indicated that the variation of gut microbial community was primarily associated with the combined effects of genetic background, age stage, and husbandry management practices. Comparative analyses identified both core and breed-specific microbial communities and revealed age-associated patterns of microbial succession. The findings provide a framework for establishing a reference profile of the boar gut microbiota and offer microbiological evidence for optimizing feeding management in commercial swine production.

## 2 Materials and methods

### 2.1 Experimental animals and fecal sample collection

This study included 1,651 commercial boars representing three major breeds (Duroc, Landrace, and Large White). Animals were recruited from three commercial boar stations operated by two large-scale livestock enterprises. Specifically, Company A operated two stations A1 and A2. Station A1 housed 34 Duroc, 113 Landrace, and 338 Large White boars, whereas the station A2 included 8 Duroc, 108 Landrace, and 301 Large White boars. Company B operated one station (B1), which housed 498 Duroc, 166 Landrace, and 85 Large White boars. The ages of experimental animals ranged from 257 to 1,906 days, covering stages from growth to maturity. The exact numbers of boars of each breed at each age stage were shown in Supplementary Table S2. All boars within the same station were maintained under identical management conditions and received the same formula diets. All experimental boars were daily inspected by trained veterinary panelists. Boars showing normal appetite, vibrant status, no respiratory symptoms, normal body temperature, and no other apparent clinical abnormalities were treated as healthy individuals and included in the further studies. Individuals receiving any antibiotics or probiotics exposure within 2 months prior to sample collection were excluded from the study. A total of 1,651 healthy boars were retained for subsequent analyses. In addition, all experimental boars were subjected to routine semen collection at a fixed interval of once every 3 days under standard and uniformed farm managements, resulting in consistent semen collection interval across the study populations. At the time of sampling, individual information including identification number, breed, age, farm, and rearing batch was recorded. Because all experimental boars were healthy, did not receive medicine and probiotics, and had the same semen collection interval, these variables were not included in the statistical models. All boars were maintained under standardized management systems within each enterprise, and the corresponding diet formulations are provided in Supplementary Table S1. Differences in husbandry practices and environmental conditions between farms were accounted for as covariates in subsequent statistical analyses.

Fecal samples were collected during the same semen collection cycle. Fresh feces samples were obtained directly from the distal rectum using sterile gloves, transferred into 2-mL sterile centrifuge tubes, and immediately flash-frozen in liquid nitrogen. Sterile sampling tools were used separately for each boar to prevent cross-contamination. After transported to the laboratory, all samples were stored at −80 °C until further use.

### 2.2 Microbial DNA extraction, metagenomic sequencing, and data processing

Microbial DNA was extracted from 1,651 samples using the Mag-Bind^®^ Soil DNA Kit (Omega Bio-tek, Norcross, GA, United States) according to manufacturer’s instructions. Concentration and purity of extracted DNA were measured using a TBS-380 (Turner BioSystems, United States) and a NanoDrop-2000 (Thermo Scientific Inc., Waltham, MA, United States), respectively. DNA quality was further checked on 1% agarose gel.

All fecal DNA samples from boars were subjected to shotgun metagenomic sequencing. DNA was fragmented to an average size of approximately 350 bp using Covaris M220 (Gene Company Limited, China) for paired-end library construction. Sequencing libraries were prepared using the NEXTFLEX Rapid DNA-Seq Kit (Bioo Scientific, Austin, TX, United States). Adapters containing the complete set of sequencing primer hybridization sites were ligated to the blunt ends of the DNA fragments. Paired-end sequencing was performed on an Illumina NovaSeq 6,000 platform (Illumina Inc., San Diego, CA, United States) using the NovaSeq 6,000 S4 Reagent Kit v1.5 (300 cycles), following to the manufacturer’s instructions at Majorbio Bio-Pharm Technology Co., Ltd. (Shanghai, China).

Paired-end Illumina reads were trimmed to remove adapter sequences, and low-quality reads (length < 50 bp or quality score < 20) were filtered using fastp (v0.23.2) (Chen et al., 2018). The resulting clean reads were then aligned to the *Sus scrofa* reference genome (Sscrofa11.1) using BWA (v0.7.9a) (Li and Durbin, 2009). Reads mapped to the host genome, together with their paired reads, were removed from further analysis. To improve the utilization rate of clean reads and reduce the computational resources and time required for sequence assembly, high-quality metagenomic sequencing reads were directly aligned to an in-house microbial reference genome database of pig gut microbiota which contained more than 10,080 species-level microbial genomes from pig guts using BWA for species detection and annotation. Alignment results were processed with CoverM (v0.7.0) (Aroney et al., 2025) to estimate taxonomic abundance (−m tpm) and coverage (−m covered_fraction). Only reads with ≥75% alignment length (−-min-read-aligned-percent 75) and ≥ 95% sequence identity (−-min-read-percent-identity 95) were retained. Effective genome coverage was calculated as reads per base (−m reads_per_base) multiplied by the average read length (151 bp). A bacterial reference genome was considered to be presence in a sample and retained for downstream analyses only when its coverage exceeded 1×. The relative abundances of microbial species were calculated using TPM (Transcripts (microbial species) Per Million).

### 2.3 Identification of shared gut microbial taxa across farms and breeds

To identify microbial taxa shared across pig farms and breeds, a presence–absence matrix was constructed based on the relative abundance data of microbial taxa from all samples. The relative abundance of >1 × 10^−5^ was used to define the as the threshold for defining the presence of a microbial species. Within each farm or breed, species with a prevalence of ≥10% were retained for subsequent analysis. Microbial species meeting these criteria were considered present in the corresponding group. Species detected in all three breeds within a single farm were defined as cross-breed shared taxa, whereas those consistently detected across farms within the same breed were defined as cross-farm shared taxa.

### 2.4 Bootstrap resampling analysis

To evaluate the robustness of the observed differences in gut microbial composition under imbalanced numbers of individuals across breeds within farms, a bootstrap-based resampling strategy was implemented. For the *α*-diversity analysis, resampling was performed within each farm. Individuals from different breeds were randomly subsampled to an equal sample size corresponding to the breed with the smallest animal number within that farm. This procedure was repeated 1,000 times to generate empirical distributions of the α-diversity metrics. For each iteration, the α-diversity indices including observed richness, Shannon, and Simpson were calculated and statistically compared between breeds using non-parametric tests. The resulting *p*-values were used to assess the stability of statistical significance. For the β-diversity, resampling was conducted across all boar populations. Individuals in each breed from all three farms were randomly subsampled to a common sample size defined by the smallest group across all breeds and farms. The resampling procedure was repeated 1,000 times. The β-diversity was evaluated based on the Bray–Curtis dissimilarity, followed by permutational multivariate analysis of variance (PERMANOVA) with 999 permutations to test the differences in the microbial community composition. The distribution of *p*-values across resampling iterations was used to determine whether the observed differences were robust to sample size imbalance. A consistent enrichment with *p* < 0.05 was evidenced as the stable and reproducible differences in the gut microbial composition.

### 2.5 PERMANOVA analysis identifying factors shaping boar gut microbial community structure

To evaluate the contribution of host and environmental factors to the variation in boar gut microbiota composition, PERMANOVA was performed using the adonis2 function in the vegan package (999 permutations, by = “terms”). Relative abundance matrices were generated after quality filtering and host-read removal of metagenomic sequencing data. Zero values were retained, and data were normalized to relative abundance prior to analysis. Explanatory variables included company, farm, breed, age, and sampling batch. Age was classified into three physiological stages (≤12 months, 12–36 months, and >36 months).

To control the effect of confound factors on the statistics of the contribution of each factor to the variation in boar gut microbial composition, an initial model was fitted across all samples in each farm (Bray–Curtis distance ~ breed + age + batch) or each age stage (Bray–Curtis distance ~ farm + breed + batch) using Bray–Curtis dissimilarity to estimate the proportion of variance in the gut microbiota composition explained (*R*^2^) by each factor. Marginal *R*^2^ values were used to compare the relative contributions of explanatory factors under different stratification conditions. Duroc pigs from the Farm A2 were excluded from this analysis because of its small sample size (*n* = 8).

### 2.6 Multivariate linear model for identifying gut microbial taxa enriched in each of three breeds and at each age stage

To identify gut microbial taxa showing stable enrichment in each of three pig breeds, as well as taxa enriched at each of three age stages, multivariable linear regression analyses were performed using MaAsLin2. Breed or age was treated as the primary fixed effect, whereas company, farm, and sampling batch were included as covariates. To identify microbial taxa enriched in each breeds, breed was alternately set as the reference category, and three pairwise models were fitted (Duroc vs. Landrace, Duroc vs. Large White, and Landrace vs. Large White) to estimate regression coefficients and corresponding significance levels for each microbial species.

To define taxa consistently enriched in a given breed relative to the other two breeds, a cross-comparison consistency strategy was applied. A species was classified as consistently enriched species if it showed concordant directions of enrichment in both pairwise comparisons against the other two breeds and reached Benjamini–Hochberg-adjusted q-values < 0.05 in both models. To identify microbial taxa enriched at each of three age stages, samples were grouped into three age stages (≤12 months, 12–36 months, and >36 months). Each age group was sequentially treated as the reference category, and all pairwise comparisons were performed (≤12 months vs. 12–36 months; ≤12 months vs. >36 months, and 12–36 months vs. >36 months). These models included the same covariates (company, farm, and batch) and applied the same multivariable regression framework and consistency criteria as that used in the breed-associated analysis. To reduce the influence of low-abundance taxa, species detected in fewer than 5% of samples or with an average relative abundance < 1 × 10^−5^ across all samples were excluded from analysis.

### 2.7 Functional annotation and pathway analysis of gut microbiota

Functional profiling of the gut microbiome was determined using HUMAnN (v3.0) (Beghini et al., 2021). Clean reads were mapped to the UniRef90 protein database to quantify gene family abundances. Gene families were subsequently grouped into metabolic pathways based on the MetaCyc database to generate pathway-level abundance profiles. In addition, KEGG Orthology (KO) annotations derived from HUMAnN were subjected to pathway enrichment analysis using the R package clusterProfiler (v4.14.6) to further characterize functional profiles of the gut microbiome (Wu et al., 2021). Gene family and pathway abundances were normalized to relative abundances to facilitate comparisons across samples, and low-abundance features were filtered prior to downstream analyses. Differentially functional pathways among boars in different breeds and at age stages were identified using linear discriminant analysis effect size (LEfSe) with an absolute LDA score > 2 and *p* < 0.05.

### 2.8 Statistical analysis

The *α*-diversity indices including observed richness, Shannon, and Simpson indices were calculated using the diversity function in the vegan package based on the standardized relative abundance matrix. For the β-diversity analysis, Bray–Curtis dissimilarities were computed from the relative abundance matrix using the vegdist function, followed by principal coordinate analysis (PCoA) for ordination and visualization. Differences in community structure between boar groups were assessed using the Wilcoxon rank-sum test, and *p*-values were adjusted for multiple comparisons using the Benjamini–Hochberg procedure. All statistical analyses were conducted in R (v4.4.2), with visualization based on the ggplot2 package (v3.5.1).

## 3 Results

### 3.1 Quality assessment of metagenomic sequencing data

**Figure 1:**
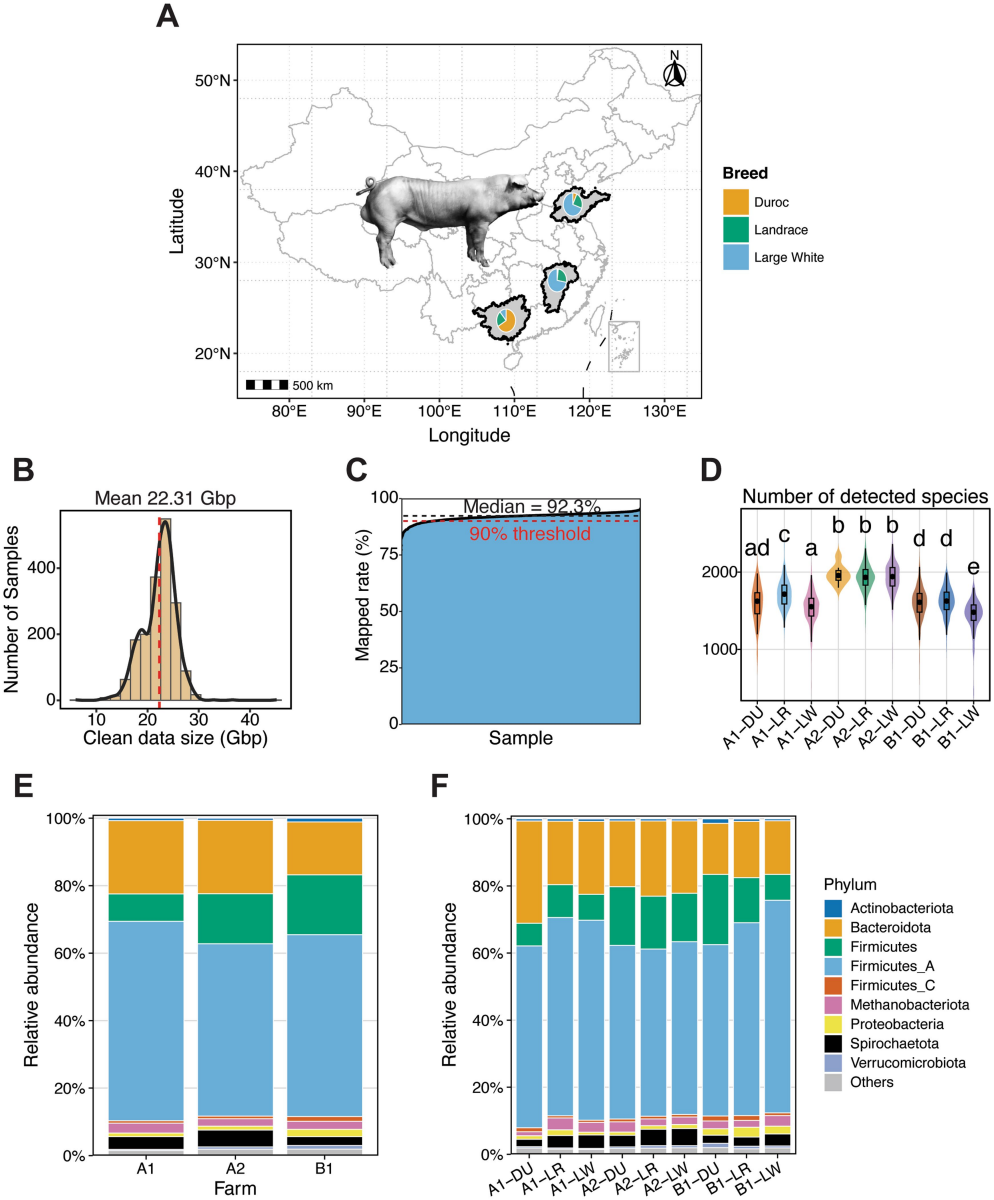
Overviews of fecal sample distribution, metagenomic sequencing depth, mapped clean read rates, and gut microbial composition. **(A)** Geographic sampling locations of boar fecal samples from three breeds (Duroc, Landrace, and Large White). **(B)** Distribution of the sizes of clean read data from 1,651 boars after quality control. An average of 22.31 Gbp clean reads per sample was obtained. **(C)** Distribution of mapped clean read rates to the microbial reference genome database. A median value of 92% mapped clean reads was obtained for 1,651 metagenomes. **(D)** Comparison of the number of detectable microbial species among three breeds within each farm. Different lowercases represent significant difference in detected microbial species. **(E)** Bar plots showing phylum-level gut microbial composition across different farms. **(F)** Bar plots showing phylum-level gut microbial composition of different boar breeds within each farm.

A total of 1,651 fecal samples from Duroc, Landrace, and Large White boars were collected from three commercial boar stations, and all samples were subjected to shotgun metagenomic sequencing (Figure 1A). The average of sequencing depth was 22.31 Gbp per sample (Figure 1B). After quality control, the number of clean reads per sample ranged from 3.9 × 10^7^ to 2.9 × 10^8^, indicating sufficient sequencing depth. The alignment rate of clean reads to the species-level reference genome database ranged from 77.27 to 95.71%, with a median alignment rate of 92.31% (Figure 1C). These results indicated high quality of sequencing data and suggested that the constructed pig gut microbial reference genome database adequately represented the gut microbial composition of pig populations used in this study.

### 3.2 Distinct gut microbial composition and diversity across nine boar groups

We compared the numbers of detected microbial species in fecal samples among three breeds. To minimize the influence of other factors, these comparisons were performed within each farm. In Farm A1, Landrace (LR) samples contained significantly more microbial species samples than Duroc (DU) and Large White (LW), whereas no significant difference was observed between DU and LW. In Farm B1, DU and LR samples harbored significantly more microbial species than LW samples. In contrast, no significant differences among three breeds were detected in Farm A2. Notably, Farm A2 exhibited the highest numbers of detected microbial species in all three pig breeds (Figure 1D and Supplementary Table S3).

At the phylum level, Firmicutes and Bacteroidota were the predominant phyla across all boar groups, constituting the core phyla of the boar gut microbiome (Figure 1E). In Farm A1, Bacteroidota showed the highest relative abundance in Duroc boars (30.4%) and the lowest relative abundance in Landrace boars (18.9%). In Farm B1, Duroc boars exhibited a lower relative abundance of Firmicutes_A (51.1%) than Large White boars (63.4%), while showing the highest relative abundance of Firmicutes (20.9%) and the lowest relative abundance of Bacteroidota (15.2%). In contrast, microbial composition in Farm A2 appeared to be relatively balance among breeds (Figure 1F). These results indicated that differences in farm environment and the genetic background of boars are associated with variations in gut microbial composition.

**Figure 2:**
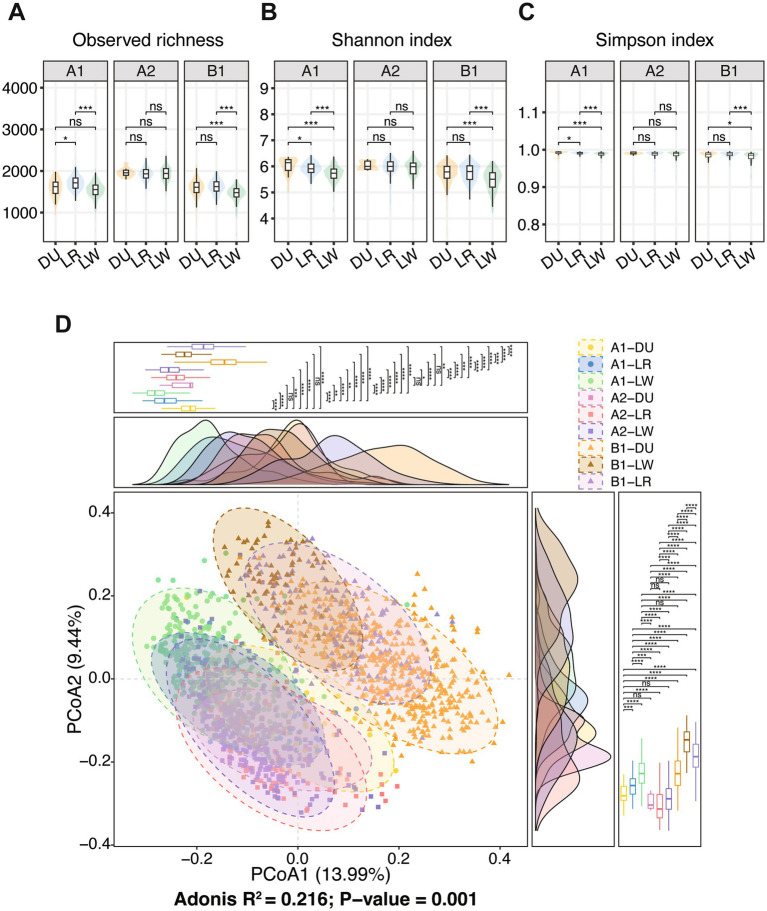
Comparison of gut microbial diversity among different boar breeds within each of three farms. Panels **(A–C)** show the comparison of *α*-diversity indices of gut microbiota among different boar breeds within each of three farms. From left to right, observed richness, Shannon index, and Simpson index are presented. Statistical significance is indicated as follows: *p < 0.05, **p < 0.01, ***p < 0.001; *ns*, not significance. **(D)** PCoA based on Bray–Curtis distances showing different *β*-diversity of the gut microbial composition among different breeds from three farms.

We next examined the gut microbial diversity among different boar groups. First, the *α*-diversity was compared among the three breeds within each farm. Significant differences in observed richness, Shannon index, and Simpson index were detected among breeds in Farms A1 and B1, whereas no significant differences were observed in Farm A2. In both Farms A1 and B1, Large White boars exhibited significantly lower α-diversity across all three indices than Duroc and Landrace boars. Within Farm A1, Duroc boars showed lower observed richness than Landrace boars but higher Shannon and Simpson indices, suggesting a more even microbial community structure in Duroc pigs (Figures 2A–C). PCoA based on the Bray–Curtis dissimilarity was subsequently conducted to assess differences in microbial community structure among boar populations. Significant separation among boar populations was observed in the ordination space (Figure 2D and Supplementary Figure 1). Fecal samples from the same farm clustered closely in the principal coordinate space, indicating greater similarity in microbial composition within farms. In addition, samples from different farms operated by the same company (e.g., A1 and A2 farms managed by Company A) were positioned closer to each other than to samples from farms operated by different companies. Although these farms were geographically separated, similarities in pig genetic background, feed formulation, and management practices within the same company may have contributed to the reduced differences in gut microbiota composition.

To evaluate the effect of the unequal sample sizes on the comparison of the gut microbial diversity among breeds within each farm, we further performed a bootstrap-based resampling analysis. Consistent with the result observed in all samples, observed richness, Shannon index, and Simpson index were significantly different among three pig breeds in both farms A1 and B1 across all resampling iterations (*p* < 0.05), and there was no significant difference among three breeds in the farm A2 (Supplementary Figure 2). For the β-diversity, PERMANOVA results across resampling iterations indicated that the different microbial community structure still existed among breeds in three farms (Supplementary Figure 3). Together, these results demonstrated that the observed differences in the gut microbial diversity among boar populations were not driven by sample size imbalance.

### 3.3 Numbers of shared or breed-specific gut microbial species among three breeds across farms

**Figure 3:**
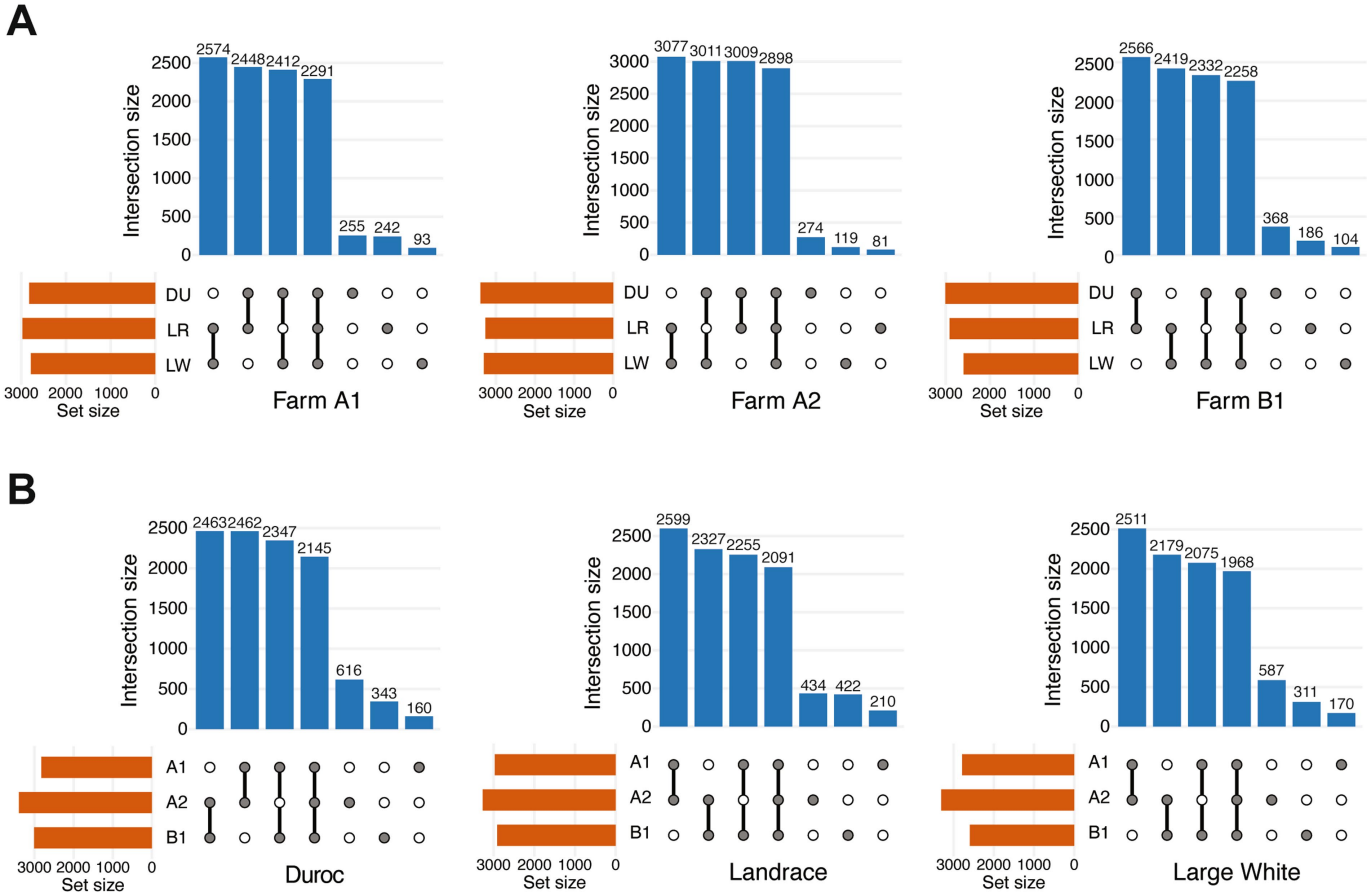
Numbers of shared or breed-specific gut microbial species among three breeds across farms. **(A)** Shared bacterial species among three pig breeds (Duroc, DU; Landrace, LR; Large White, LW) within the same farm. From left to right, the data of farms A1, A2, and B1 are shown. **(B)** Shared bacterial species of the same pig breed across three different farms (A1, A2, and B1). From left to right, the data for Duroc, Landrace, and Large White pigs are shown. In the UpSet plots, connecting lines indicate shared relationships; the upper bars and corresponding numbers represent the number of shared species, while the left bars indicate the total number of species detected in each set.

To evaluate microbial species shared among the three breeds under different rearing environments, species-level overlap analyses were performed within each farm. Using a uniform abundance threshold criteria, the number of species shared among breeds within the same farm was quantified by identifying taxa consistently present in all three breeds. Approximately 3,000 species were detected in all samples. Samples from Farm A2 showed the highest number of microbial species (2,898) shared by all three breeds. In both Farms A1 and A2, the number of shared species between Landrace and Large White exceeded the numbers that observed in the comparisons involving Duroc, indicating greater microbial similarity between these two breeds under the same rearing conditions. In both Farms A1 and B1, Duroc and Large White shared the fewest number of microbial species, whereas at Farm A2, Duroc and Landrace showed the lowest overlap of microbial species. Regarding breed-specific taxa, Duroc exhibited the highest number of unique species in all three farms (255, 274, and 368 species in the farm A1, A2, and B1, respectively). In contrast, Large White showed fewer unique species across all farms (93, 119, and 104 species, respectively) (Figure 3A). These results indicated that the extent of inter-breed microbial species varied across rearing environments and that Duroc pigs exhibited relatively greater microbial specificity.

Subsequently, to evaluate the effect of rearing environments on the gut microbial structure, we analyzed the number of species shared among different farms within each breed. Using the same abundance thresholds and prevalence filtering criteria, microbial species consistently detected across farms were identified. Each breed harbored a subset of core microbiota shared among farms, with Duroc showing the highest number of shared species (2,145 species) across farms (Figure 3B). For Landrace and Large White, the number of shared species between the two farms operated by Company A (A1 and A2) were greater than those shared Company B (B1). Moreover, the lowest number of shared microbial species were observed between A1 and B1 farms in all three breeds. This finding was consistent with the results observed in the PCoA. Regarding farm-specific taxa within each breed, Farm A2 exhibited the highest numbers of unique species in all three breeds (616, 434, and 587 species, respectively), whereas Farm A1 showed the lowest numbers (160, 210, and 170 species). These results indicated that the variation in rearing environments and management systems was associated with divergent gut microbial composition within the same breed.

### 3.4 Multiple factors drove the variations in the composition of gut microbiota among experimental boar populations

**Figure 4:**
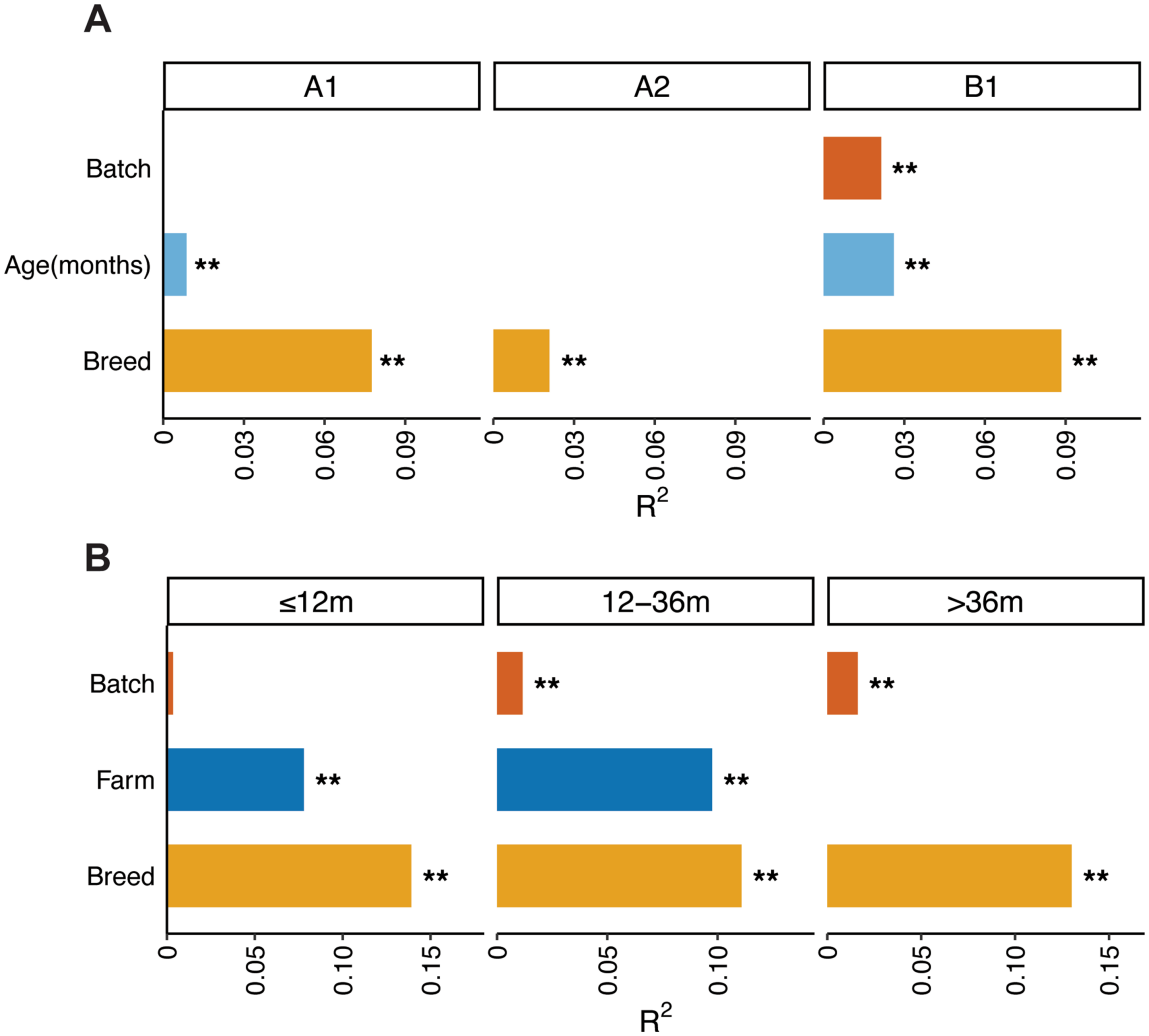
Contribution of different factors to the variation of gut microbial composition in boars within each farm and each age stage. **(A)** The contribution of various factors in the variation of gut microbial composition within each farm. (A1, A2, and B1 represent different farms). **(B)** The contribution of each factor in the variation of gut microbial composition at each of three age stages (≤12 months, 12–36 months, and >36 months). Bar length indicates the proportion of gut microbial composition variation (*R*^2^) explained by each factor based on PERMANOVA using Bray–Curtis distance matrices. Asterisks denote significance levels (**p < 0.01).

PERMANOVA analyses were performed to evaluate the contribution of various factors including breed, age, batch, and farm to the variation in boar gut microbial composition. The results showed that the contributions of explanatory factors to gut microbial structure varied in different farms. Breed was significantly contributed to the variation in boar gut microbial composition in all farms. The largest proportion of explained variance was observed in Farm B1 (*R*^2^ = 9%), followed by Farm A1 (*R*^2^ = 8%) and Farm A2 (*R*^2^ = 2%). In Farm A1, age showed a weak but still significant contribution (*R*^2^ = 1%). Breed and age together explained approximately 9% of the variation in boar gut microbial composition. In Farm A2, breed explained approximately 2% of the variation in the gut microbial composition. In Farm B1, breed, age, and batch together explained approximately 14% of the variance, including 9% of variance to breed, 3% to age, and 2% to batch. Although batch accounted for only a small proportion of the variance, its effect remained statistically significant in Farm B1 (Figure 4A).

Given that the effect size of each host and environmental factor on boar gut microbial composition varied at different age stage, we further evaluated the contributions of multiple factors to variations in boar gut microbial composition at each of three developmental stages. In young boars (≤12 months), breed accounted for the largest proportion of variation in gut microbiota composition (*R*^2^ = 13.9%), followed by farm (*R*^2^ = 7.8%), whereas sampling batch showed no significant effect. In boars at the age of 12–36 months, breed remained the main contributor to the variation of boar gut microbial composition (*R*^2^ = 11.1%), followed by farm (*R*^2^ = 9.8%). However, sampling batch only contributed 1.2% of the variance. In aged boars (>36 months), breed and sampling batch explained 13.0 and 1.6% of the variation in gut microbial composition, respectively (Figure 4B). Overall, host genetics and farm explained large proportions of the variations in the gut microbial composition across three age stages.

### 3.5 Gut microbial taxa enriched in the boar gut of each breed

**Figure 5:**
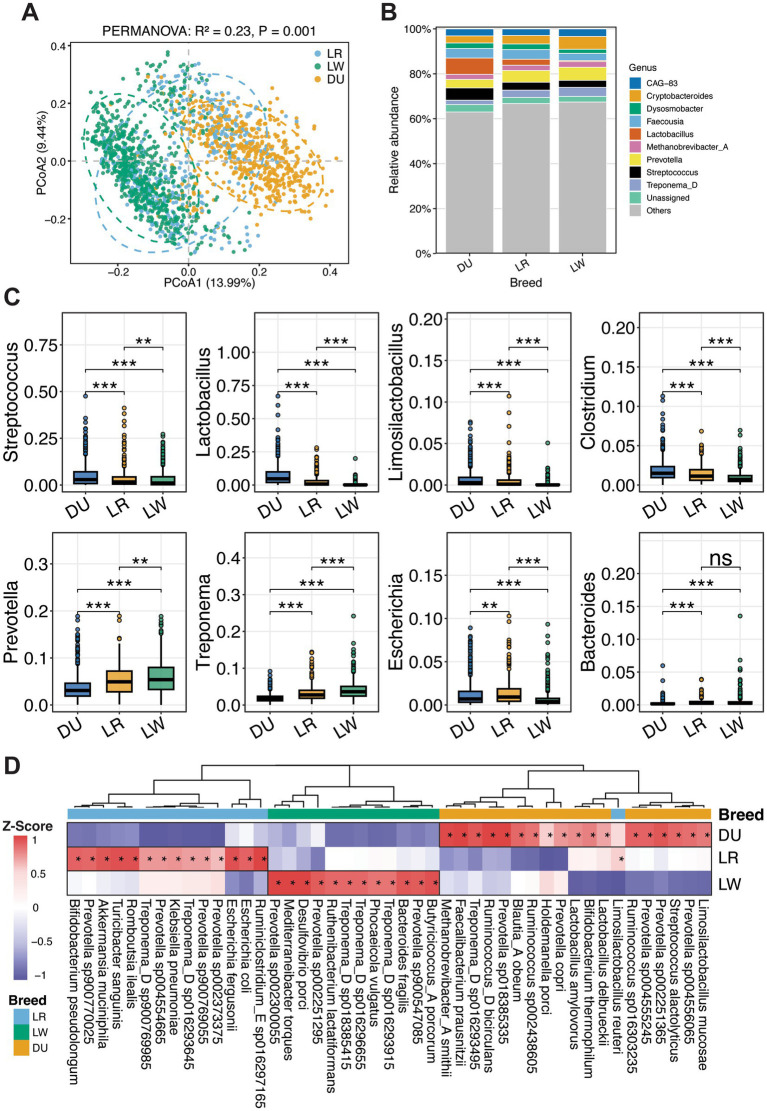
Difference in the gut microbiota compositions among three pig breeds. **(A)** PCoA based on Bray–Curtis distances demonstrating significant differences in the gut microbial composition among three breeds. **(B)** Stacked bar plots at the genus level showing the gut microbial composition in three pig breeds of Duroc (DU), Landrace (LR), and Large White (LW). **(C)** Comparison of the relative abundances of eight core genera among different breeds. Significance is indicated by asterisks (*p < 0.05, **p < 0.01, and ***p < 0.001). **(D)** Gut microbial taxa enriched in the boar gut of each breed. Asterisks indicate significantly enriched taxa in the corresponding group. Colors represent *Z*-score–transformed relative abundance values, with red indicating higher abundance and blue indicating lower abundance.

We then identified gut microbial taxa enriched in each boar breed using multivariable association analysis with MaAsLin2. The model incorporated farm, breed, age, and sampling batch as covariates to estimate the independent effect of breed on genus- and species-level abundance. Significant differences in gut microbial composition were observed among the three boar breeds (Figure 5A). At the genus level, *Streptococcus*, *Lactobacillus*, *Limosilactobacillus*, and *Clostridium* showed the highest relative abundances in Duroc pigs. Interestingly, lactic acid bacteria were enriched in Duroc pigs with *Lactobacillus* reaching an average relative abundance of 7.3% (2.7 and 0.5% in Landrace and Large White pigs, respectively). In contrast, *Prevotella* and *Treponema* were more abundant in Large White, with average relative abundances of 5.8 and 4.0%. Most genera displayed intermediate abundance levels in Landrace. However, *Escherichia* exhibited the highest abundance in Landrace pigs, followed by Duroc pigs. *Bacteroides* showed comparable relative abundances in Landrace and Large White pigs, but was significantly lower in Duroc pigs (Figures 5B,C).

At the species level, we focused on those microbial species significantly enriched in a given breed in pairwise comparisons (*P_adj_* < 0.05). The gut microbiota of Duroc boars was characterized by stable enrichment of *Lactobacillus amylovorus*, *Limosilactobacillus mucosae*, *Streptococcus alactolyticus*, *Faecalibacterium prausnitzii*, *Ruminococcus_D bicirculans*, *Blautia_A obeum*, *Holdemanella porci*, and *Bifidobacterium thermophilum*, together with the hydrogenotrophic archaeon *Methanobrevibacter_A smithii*. These taxa have been reported to participate in carbohydrate fermentation and short-chain fatty acid production (Figure 5D and Supplementary Table S4). The gut microbiota enriched in Landrace boars included *Prevotella* spp., *Akkermansia muciniphila*, *Lactobacillus reuteri*, *Turicibacter sanguinis*, *Romboutsia ilealis*, and *Bifidobacterium pseudolongum*, as well as members of the Enterobacteriaceae, including *Escherichia coli*, *Escherichia fergusonii*, and *Klebsiella pneumoniae* (Figure 5D and Supplementary Table S4). This gut microbial structure was characterized by the coexistence of potentially beneficial and potentially pathogenic bacteria. In Large White boars, the gut microbiota was enriched in taxa associated with the degradation of host-derived mucus and complex polysaccharides, including *Bacteroides fragilis*, *Prevotella* spp., *Treponema_D* spp., *Mediterraneibacter torques*, *Phocaeicola vulgata*, *Ruthenibacterium lactatiformans*, *Butyricicoccus_A porcorum*, and *Desulfovibrio porci* (Figure 5D and Supplementary Table S4).

### 3.6 Age-specific enrichment of gut microbial taxa in boars

**Figure 6:**
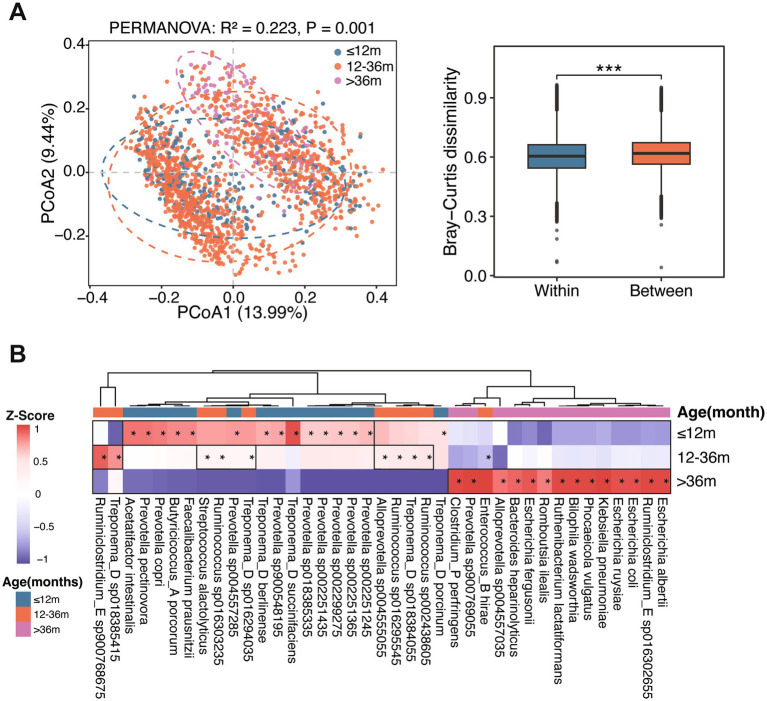
Age-associated enrichment patterns of gut microbiota in boars. **(A)** Left: The PCoA showing significant differences in gut microbial community structure across three age stages (≤12 months, 12–36 months, and >36 months). Right: Boxplot of within-group and between-group differences based on Bray–Curtis distances, with significance indicated by asterisks (*p < 0.05, **p < 0.01, and ***p < 0.001). **(B)** Age-specific enrichment of gut microbial taxa in boars. Gut microbial composition were analyzed at the age of ≤12 months, 12–36 months, and >36 months. Asterisks indicate significantly enriched taxa in the corresponding group. Colors represent *Z*-score–transformed relative abundance values, with red indicating higher abundance and blue indicating lower abundance.

Significant differences were observed in gut microbial community structure among the three age stages (Figure 6A). To characterize age-associated dynamics in gut microbial composition, we constructed multivariable models to identify microbial taxa enriched at each of the three age stages. Farm, breed, and sampling batch were included as covariates. The analysis revealed age-dependent shifts in gut microbial composition across the three age stages. In young boars, the gut microbiota was characterized by the enrichment of polysaccharide-fermenting bacteria, which mainly included *Prevotella copri*, *Prevotella pectinovora*, *Prevotella sp002251435*, *Treponema berlinense*, *Treponema succinifaciens*, and *Treponema porcinum*. These taxa harbored diverse polysaccharide utilization loci and glycoside hydrolases, supporting the degradation of complex carbohydrates, such as hemicellulose and pectin, to produce SCFAs via the succinate–propionate pathway. Concurrently, butyrate-producing bacteria, including *Butyricicoccus porcorum*, *Faecalibacterium prausnitzii*, and *Acetatifactor intestinalis*, were also enriched in the gut of young boars (Figure 6B and Supplementary Table S5).

At the age of 12–36 months, the gut microbiota was enriched in fiber-degrading bacteria, including *Ruminococcus* spp., *Treponema* spp., and *Ruminiclostridium_E sp900768675*, and by lactate-producing bacteria, such as *Streptococcus alactolyticus* and *Alloprevotella sp004555055* (Figure 6B and Supplementary Table S5). In the aged stage, the gut microbiota showed higher abundances of *Ruthenibacterium lactatiformans*, *Romboutsia ilealis*, *Bilophila wadsworthia*, *Bacteroides heparinolyticus*, and *Phocaeicola vulgatus* than those in the other two ages (Figure 6B and Supplementary Table S5). Among these taxa, *R. lactatiformans* and *R. ilealis* were associated with amino acid and peptide fermentation (Shkoporov et al., 2016; Gerritsen et al., 2017). *B. wadsworthia* utilized taurine derived from taurine-conjugated bile acids for sulfite respiration and hydrogen sulfide production (Carbonero et al., 2012; Burrichter et al., 2021; Sayavedra et al., 2025). In addition, increased relative abundances of *Escherichia* spp., including *E. coli*, *E. albertii*, and *E. fergusonii*, together with *Klebsiella pneumoniae* and *Clostridium perfringens*, were observed, potentially indicating altered barrier homeostasis and an increased risk of pathogens expansion in aged boars.

### 3.7 Potential functional profiles of boar gut microbiome across breeds and age stages

Based on HUMAnN annotation, we compared the functional pathway profiles of gut microbiome across boar breeds and revealed marked divergence in potential functional capacities among three breeds (Supplementary Figure 4). In Duroc boars, enriched pathways were mainly associated with protein synthesis and cell proliferation, including ribosome, aminoacyl-tRNA biosynthesis, DNA replication, and DNA repair, as well as pathways related to carbohydrate transport and utilization, such as starch and sucrose metabolism, and phosphotransferase system. In addition, pathways related to cell wall biosynthesis, including peptidoglycan and teichoic acid biosynthesis, were also enriched in the gut microbiome of Duroc boars, which might suggest the relatively active microbial growth and substrate utilization potential. The gut microbiome of Landrace boars showed enrichment of pathways related to environmental adaptation and host interaction, including flagellar assembly, bacterial chemotaxis, and biofilm formation. Lipopolysaccharide biosynthesis and glutathione metabolism were also significantly enriched in Landrace boars, which might suggest a functional profile potentially associated with microbial stress adaptation and host–microbe interaction. This enrichment was possibly related to the high abundance of Enterobacteriaceae. Pathways enriched in Large White boars were mainly related to central carbon and energy metabolism, including glycolysis/gluconeogenesis, the tricarboxylic acid cycle, pentose phosphate pathway, and pyruvate metabolism. Multiple amino acid metabolism pathways and short-chain fatty acid-related pathways, such as arginine biosynthesis, butanoate, and propanoate metabolism, were also significantly enriched in the gut microbiome of Large White boars. All these suggested an enhanced potential for the degradation and utilization of complex substrates. This was consistent with the enrichment of polysaccharide-degrading taxa observed in this breed although further experimental validation would be needed.

We further observed significant differences in functional pathway profiles of the gut microbiome across age stages in boars (Supplementary Figure 5). In young boars, the gut microbiome was enriched in central carbon metabolism and amino acid biosynthesis pathways, including glycolysis/gluconeogenesis, citrate cycle, pyruvate metabolism, pentose phosphate pathway, and biosynthesis of amino acids, together with enhanced metabolism of branched-chain and sulfur-containing amino acids. In addition, pathways related to cofactor and vitamin metabolism were also significantly enriched in the gut microbiome of young boars, suggesting its biosynthesis and utilization potential of vitamins at this age stage. This was also consistent with the growing demands of vitamins for this age stage. In boars at the age of 12–36 months, the pathways mainly involved in protein synthesis and basic metabolic regulation, including aminoacyl-tRNA biosynthesis, amino sugar and nucleotide sugar metabolism, glycine, serine and threonine metabolism, and fatty acid biosynthesis, were enriched in their gut microbiome. These results suggested a functional shift of bacteria from rapid growth toward the maintenance of cellular structure and metabolic homeostasis. In contrast, the gut microbiome of aged boars exhibited a distinct functional profile. Pathways related to environmental adaptation and stress response, including quorum sensing, biofilm formation, bacterial secretion system, flagellar assembly, and antimicrobial resistance pathways, were enriched. Consistent with the age-associated enrichment of opportunistic pathogens in the gut microbiome of aged boars, the pathways of lipopolysaccharide biosynthesis, peptidoglycan biosynthesis, and oxidative phosphorylation were significantly enriched in the gut microbiome of aged boars, possibly suggesting enhanced microbial stress adaptation and a potentially altered community functional profile.

## 4 Discussion

Boars are critical to pig production, as they directly influence the productivity and efficiency of the swine industry. However, whether the gut microbiota influences boar reproductive traits, such as semen production and quality is largely unknown. Understanding the gut microbial composition and its influencing factors in large-scale boar populations is an essential first step toward addressing this question. In this study, we analyzed the gut microbial composition in more than 1,650 boars from three popular commercial breeds and investigated the effects of environmental and host factors on gut microbial composition. The results provided an important foundation for deeply investigating the effect of gut microbiota on reproduction traits of boars and for regulating gut microbiota to improve boar health and production traits in the future.

We observed significant difference in gut microbial composition among nine boar populations representing three pig breeds across three farms. This finding indicated that environmental and host factors jointly shaped the composition and functional potential of the porcine gut microbiota. Breed, age, and rearing environment were all significantly contributed to the variations of the gut microbial compositions, with breed explaining 2% ~ 9% of the variance in the gut microbial composition. This finding is consistent with previous reports showing that host genetics contributes to inter-individual differences in microbial composition and the abundance of specific taxa, although its overall contribution to community-level variation remains limited (Bonder et al., 2016; Turpin et al., 2016; Kurilshikov et al., 2021; Sanna et al., 2022). Large-scale human population studies have estimated that host genetic variation explains approximately 1.9 to 8.1% of overall microbiome variation, although the heritability of certain bacterial taxa could reach 0.2 to 0.4 within specific lineages (Goodrich et al., 2016; Lopera-Maya et al., 2022). This range of explained variation was comparable to that observed in our study. Microbiome genome-wide association studies (mGWS) have further identified *LCT* and *ABO* that are associated with specific gut microbial taxa (Kurilshikov et al., 2021); (Yang et al., 2022). Together, these findings suggested that host genetic variants may shape microbial ecological niches by regulating the availability of intestinal metabolic substrates.

Environmental factors also play an important role in shaping the boar gut microbiota. In this study, environmental variables (farms) explained a proportion of the variation in gut microbial composition that was comparable to that explained by breed. Among these variables, diet has been widely recognized as a major driver of microbial community dynamics (den Besten et al., 2013; Rowland et al., 2017; Zhang et al., 2020). Previous studies have shown that dietary interventions can substantially alter gut microbiota and its metabolic pathways, including pathways related to bile acid metabolism, amino acid fermentation, and short-chain fatty acid production (David et al., 2013). Rothschild et al. (2018) reported that gut microbiota composition was not significantly associated with host genetic ancestry, whereas environmental factors such as diet, medication use, and lifestyle accounted for more than 20% of the inter-individual variation in the gut microbiota. Similarly, PCoA analysis showed clear clustering of samples from the same farm, and boars within the same farm shared more bacterial taxa.

Distinct microbial community structures were observed among boars of different breeds. In Duroc boars, the enrichment of *Lactobacillus*, *Bifidobacterium*, and several butyrate-producing taxa possibly suggested the presence of a fermentative microbial consortium that might promote SCFAs production through lactate–butyrate cross-feeding, thereby potentially supporting the efficient energy utilization and metabolic stability within the intestinal ecosystem (Lebas et al., 2020; Zhao et al., 2024). These microbial features are consistent with the possibility that long-term genetic selection for growth performance and feed efficiency in Duroc pigs could have contributed to the establishment of a gut microbial community with enhanced fermentative capacity (Ding et al., 2018; Fernández-Veledo and Vendrell, 2019). In contrast, the gut microbiota of Landrace boars showed an increased abundance of *A. muciniphila* and *L. reuteri*, which might possibly be associated with enhanced mucin turnover and host–microbiota interactions (Kiššová et al., 2022; Luo et al., 2022). Meanwhile, the enrichment of Enterobacteriaceae may reflect an adaptive response to nutrient-rich diets with elevated protein or energy levels (Beam et al., 2021; Gouveia et al., 2024). We further observed relatively lower species richness in the gut microbiota of Large White boars than in the other two pig breeds, particularly in the Farm B1. This pattern may reflect the combined effects of host genetic background and environmental conditions. The gut microbial community of Large White boars was dominated by *Bacteroides*, *Prevotella*, and *Treponema*, have been associated with complex polysaccharide degradation and propionate or succinate metabolism (Fernández-Veledo and Vendrell, 2019; Vishnu Prasoodanan et al., 2021). In addition, the concurrent enrichment of butyrate-producing bacteria, including *M. torques* and *B. porcorum* possibly improved the energy reutilization and the maintenance of epithelial homeostasis (Trachsel et al., 2018; Venegas et al., 2019; Lou et al., 2025). Overall, these breed-associated microbial patterns may reflect the combined influences of long-term host genetic selection and dietary differences among breeds.

In mammals, the gut microbiota undergoes pronounced age-associated dynamics and is closely linked to changes in diet, intestinal physiology, and the immune system (Yatsunenko et al., 2012). Evidence from swine production systems indicates that age is a major determinant of gut microbiome variation. In particular, the weaning transition induces rapid and transient shifts in microbial composition, followed by progressive stabilization during the late finishing stage (Chen et al., 2017; Sciellour et al., 2019; Wang et al., 2019). Longitudinal studies have further demonstrated that the porcine gut microbiota exhibits clear age-dependent dynamics across developmental stages, accompanied by coordinated changes in community structure and functional potential (Wang et al., 2019; Ma et al., 2022). However, these studies have generally relied on small longitudinal cohorts or focused primarily on early growth stages, and population-level analyses in commercial boars remain limited. In the present study, we identified age-associated enrichment patterns of the gut microbiota across different developmental stages in boars using multivariable models. *Prevotella* spp. and *Treponema* spp., which are known to harbor extensive polysaccharide utilization loci and glycoside hydrolase systems, were enriched in young boars. These taxa may facilitate the degradation of plant-derived complex polysaccharides (Cwyk and Canale-Parola, 1979; Gálvez et al., 2020). Concurrently, early enrichment of butyrate-producing bacteria, such as *F. prausnitzii*, might possibly be linked to the energy supplement to the intestinal epithelium and immune regulation that contributes to intestinal homeostasis (Lopez-Siles et al., 2017; Lenoir et al., 2020). In adult boars, fiber-degrading taxa, including *Ruminococcus* spp. were co-enriched with lactic acid-producing bacteria such as *S. alactolyticus*. Lactate utilization by butyrate-producing bacteria for short-chain fatty acid production may indicate enhanced microbial utilization of structural carbohydrates at this stage (Culp and Goodman, 2023). In contrast, aged boars showed an enrichment of several opportunistic pathogens, including *E. coli*, *K. pneumoniae*, and *C. perfringens*. This suggested an age-associated shift in the gut microbial composition that are potentially consistent with impaired intestinal homeostasis and a higher inflammatory risk (Guo et al., 2013). Nevertheless, these inferences require further validation through gut metabolomic analyses or *in vitro* experiments.

Previous studies have demonstrated that the gut microbiota is linked to pig production traits, such as feed efficiency and fat deposition (Bergamaschi et al., 2020; Chen et al., 2021). Increasing evidences have also supported the existence of a “gut–testis axis,” suggesting that the gut microbiota may influence spermatogenesis through multiple ways, including microbial metabolites, host immune responses, and endocrine signaling (Ding et al., 2020; Kaltsas et al., 2025). For instances, Lin et al. (2022) reported that dietary fiber supplementation reshaped the gut microbial structure of boars and increased short-chain fatty acid levels, thereby promoting spermatogenesis and improving semen quality (Lin et al., 2022). In Duroc boars, taxifolin intervention was likewise associated with an increase in beneficial bacteria and a reduction in potentially harmful taxa, accompanied by coordinated improvements in blood metabolite profiles and semen quality (Zhou et al., 2022). Furthermore, Guo et al. (2020) found significant differences in gut microbial structure and potential function capacities between boars with different semen utilization rates, and the individuals with low semen utilization exhibited impaired intestinal barrier function and elevated systemic inflammation. Han et al. (2022) further demonstrated that alginate oligosaccharide supplementation could sustainably enhance semen quality by modulating the gut microbiota and improving host metabolism. These previous findings suggested that the differences in the gut microbiota compositions observed in the present study might be potentially linked to the variations in the semen quality of boars, so the results obtained in this study should provide the foundation for further investigating the association of the gut microbiota with boar semen quality.

In summary, we investigated the composition and diversity of gut microbiota in a large-scale cohort of 1,651 boars. Distinct microbial features were observed across breeds and age stages. We further evaluated the contributions of various host and environmental factors to variations in boar gut microbial composition, and identified microbial species enriched in each breed as well as those enriched at each of the three age stages. To our knowledge, this study is the first to systematically characterize the boar gut microbial profiles and evaluate the factors influencing gut microbial composition in a large-scale boar population. There were several limitations in this study, mainly including that: (1) this study only focused on the gut microbial composition, and no metabolomic data were available to validate the functional capacities of microbial taxa enriched in different boar breeds. (2) Although major confounders were included in the multivariable models, the influence of other unmeasured sources of environmental heterogeneity could not be fully excluded. Nevertheless, our findings provided a valuable foundation for improving boar health and management through targeted interventions on gut microbiota and for further exploring the potential associations between gut microbiota and boar reproductive traits.

## Data Availability

The metagenomic sequencing data generated in this study have been submitted to the China National GeneBank DataBase (CNGBdb) under accession code CRA039313 and are available at https://ngdc.cncb.ac.cn/gsa/browse/CRA039313.

## Supplementary material

SUPPLEMENTARY FIGURE 1 Boxplot showing within-breed and between-breed differences based on Bray–Curtis distances corresponding to the PCoA in Figure 2D. Statistical significance is indicated by asterisks (**P* < 0.05, ***P* < 0.01, and ****P* < 0.001).

SUPPLEMENTARY FIGURE 2 Distribution of *P*-values from the comparison of the α-diversity among breeds across 1,000 bootstrap-based resampling iterations within each farm. To control for unequal sample sizes among breeds within farms, individuals from each breed were randomly subsampled to the size of the smallest breed group in the corresponding farm, and the procedure was repeated 1,000 times. Histograms show the distributions of P-values obtained from Kruskal–Wallis tests for Observed richness **(A)**, Shannon index **(B)**, and Simpson index **(C)** in A1, A2, and B1 farms. The red dashed line indicates the significance threshold at *P* = 0.05.

SUPPLEMENTARY FIGURE 3 Distribution of PERMANOVA results for β-diversity across 1,000 resampling iterations. Individuals in each breed from all three farms were randomly subsampled to a common sample size defined by the smallest group across all breeds and farms, and the analysis was repeated 1,000 times. **(A)** Histogram of *P*-values obtained from PERMANOVA across resampling iterations. The red dashed line indicates the significance threshold at *P* = 0.05. **(B)** Distribution of the corresponding *R*² values, representing the proportion of variance in microbial community structure explained by group differences. The consistent enrichment of significant *P*-values together with stable *R*² distributions indicates that the observed differences in community composition are robust to sample size normalization.

SUPPLEMENTARY FIGURE 4 Differentially enriched functional pathways of the gut microbiome among three pig breeds identified by LEfSe analysis. Pathway abundance profiles derived from HUMAnN annotation were used to identify differentially enriched metabolic pathways among Duroc, Landrace, and Large White boars. The *x*-axis indicates the LDA score, and the *y*-axis shows the pathway names. Bar colors represent the breed in which each pathway was enriched, with blue for Landrace, green for Large White, and orange for Duroc. The size of the open circles indicates the significance level of each pathway, expressed as -log10(*P*-value). Pathways within each breed are ordered from high to low according to the LDA score.

SUPPLEMENTARY FIGURE 5 Differentially enriched functional pathways of the gut microbiome across three age stages identified by LEfSe analysis. Pathway abundance profiles derived from HUMAnN annotation were used to identify differentially enriched metabolic pathways among young, mature, and aged boars. The *x*-axis indicates the LDA score, and the *y*-axis shows the pathway names. Bar colors represent the age stage in which each pathway was enriched, with blue for young boars, orange for mature boars, and pink for aged boars. The size of the open circles indicates the significance level of each pathway, expressed as -log10(*P*-value). Pathways within each age stage are ordered from high to low according to the LDA score.

## References

[ref1] Aitken R. J. (2023). Male reproductive ageing: a radical road to ruin. Hum. Reprod. 38, 1861–1871. doi: 10.1093/humrep/dead157, 37568254 PMC10546083

[ref2] Allrich R. D. Christenson R. K. Ford J. J. Zimmerman D. R. (1983). Pubertal development of the boar: age-related changes in testicular morphology and in vitro production of testosterone and estradiol-17 beta. Biol. Reprod. 28, 902–909. doi: 10.1095/biolreprod28.4.902, 6860745

[ref3] Almeida F. F. L. Leal M. C. França L. R. (2006). Testis morphometry, duration of spermatogenesis, and spermatogenic efficiency in the wild boar (*Sus scrofa scrofa*). Biol. Reprod. 75, 792–799. doi: 10.1095/biolreprod.106.053835, 16870941

[ref4] Aroney S. T. N. Newell R. J. P. Nissen J. N. Camargo A. P. Tyson G. W. Woodcroft B. J. (2025). CoverM: read alignment statistics for metagenomics. Bioinformatics 41:btaf147. doi: 10.1093/bioinformatics/btaf147, 40193404 PMC11993303

[ref5] Bäckhed F. Ley R. E. Sonnenburg J. L. Peterson D. A. Gordon J. I. (2005). Host-bacterial mutualism in the human intestine. Science 307, 1915–1920. doi: 10.1126/science.1104816, 15790844

[ref6] Beam A. Clinger E. Hao L. (2021). Effect of diet and dietary components on the composition of the gut microbiota. Nutrients 13:2795. doi: 10.3390/nu13082795, 34444955 PMC8398149

[ref7] Beghini F. McIver L. J. Blanco-Míguez A. Dubois L. Asnicar F. Maharjan S. . (2021). Integrating taxonomic, functional, and strain-level profiling of diverse microbial communities with bioBakery 3. eLife 10:e65088. doi: 10.7554/eLife.65088, 33944776 PMC8096432

[ref8] Bergamaschi M. Tiezzi F. Howard J. Huang Y. J. Gray K. A. Schillebeeckx C. . (2020). Gut microbiome composition differences among breeds impact feed efficiency in swine. Microbiome 8:110. doi: 10.1186/s40168-020-00888-9, 32698902 PMC7376719

[ref9] Bhattacharya I. Dey S. Banerjee A. (2023). Revisiting the gonadotropic regulation of mammalian spermatogenesis: evolving lessons during the past decade. Front. Endocrinol. 14:1110572. doi: 10.3389/fendo.2023.1110572, 37124741 PMC10140312

[ref10] Bonder M. J. Kurilshikov A. Tigchelaar E. F. Mujagic Z. Imhann F. Vila A. V. . (2016). The effect of host genetics on the gut microbiome. Nat. Genet. 48, 1407–1412. doi: 10.1038/ng.3663 27694959

[ref11] Brooks A. W. Kohl K. D. Brucker R. M. Opstal E. J. V. Bordenstein S. R. (2016). Phylosymbiosis: relationships and functional effects of microbial communities across host evolutionary history. PLoS Biol. 14:e2000225. doi: 10.1371/journal.pbio.2000225, 27861590 PMC5115861

[ref12] Burrichter A. G. Dörr S. Bergmann P. Haiß S. Keller A. Fournier C. . (2021). Bacterial microcompartments for isethionate desulfonation in the taurine-degrading human-gut bacterium *Bilophila wadsworthia*. BMC Microbiol. 21:340. doi: 10.1186/s12866-021-02386-w, 34903181 PMC8667426

[ref13] Carbonero F. Benefiel A. C. Alizadeh-Ghamsari A. H. Gaskins H. R. (2012). Microbial pathways in colonic sulfur metabolism and links with health and disease. Front. Physiol. 3:448. doi: 10.3389/fphys.2012.00448, 23226130 PMC3508456

[ref14] Chen C. Fang S. Wei H. He M. Fu H. Xiong X. . (2021). *Prevotella copri* increases fat accumulation in pigs fed with formula diets. Microbiome 9:175. doi: 10.1186/s40168-021-01110-0, 34419147 PMC8380364

[ref15] Chen L. Xu Y. Chen X. Fang C. Zhao L. Chen F. (2017). The maturing development of gut microbiota in commercial piglets during the weaning transition. Front. Microbiol. 8:1688. doi: 10.3389/fmicb.2017.01688, 28928724 PMC5591375

[ref16] Chen S. Zhou Y. Chen Y. Gu J. (2018). Fastp: an ultra-fast all-in-one FASTQ preprocessor. Bioinformatics 34, i884–i890. doi: 10.1093/bioinformatics/bty560, 30423086 PMC6129281

[ref17] Culp E. J. Goodman A. L. (2023). Cross-feeding in the gut microbiome: ecology and mechanisms. Cell Host Microbe 31, 485–499. doi: 10.1016/j.chom.2023.03.016, 37054671 PMC10125260

[ref18] Cwyk W. M. Canale-Parola E. (1979). *Treponema succinifaciens* sp. nov., an anaerobic spirochete from the swine intestine. Arch. Microbiol. 122, 231–239. doi: 10.1007/bf00411285, 120726

[ref19] David L. A. Maurice C. F. Carmody R. N. Gootenberg D. B. Button J. E. Wolfe B. E. . (2013). Diet rapidly and reproducibly alters the human gut microbiome. Nature 505, 559–563. doi: 10.1038/nature12820, 24336217 PMC3957428

[ref20] den Besten G. van Eunen K. Groen A. K. Venema K. Reijngoud D. J. Bakker B. M. (2013). The role of short-chain fatty acids in the interplay between diet, gut microbiota, and host energy metabolism. J. Lipid Res. 54, 2325–2340. doi: 10.1194/jlr.R036012, 23821742 PMC3735932

[ref21] Ding R. Yang M. Wang X. Quan J. Zhuang Z. Zhou S. . (2018). Genetic architecture of feeding behavior and feed efficiency in a duroc pig population. Front. Genet. 9:220. doi: 10.3389/fgene.2018.00220, 29971093 PMC6018414

[ref22] Ding N. Zhang X. Zhang X. D. Jing J. Liu S. S. Mu Y. P. . (2020). Impairment of spermatogenesis and sperm motility by the high-fat diet-induced dysbiosis of gut microbes. Gut 69, 1608–1619. doi: 10.1136/gutjnl-2019-319127, 31900292 PMC7456731

[ref23] Fernández-Veledo S. Vendrell J. (2019). Gut microbiota-derived succinate: friend or foe in human metabolic diseases? Rev. Endocrine Metabol. Disord. 20, 439–447. doi: 10.1007/s11154-019-09513-z, 31654259 PMC6938788

[ref24] Gálvez E. J. C. Iljazovic A. Amend L. Lesker T. R. Renault T. Thiemann S. . (2020). Distinct polysaccharide utilization determines interspecies competition between intestinal Prevotella spp. Cell Host Microbe 28, 838–852.e6. doi: 10.1016/j.chom.2020.09.012, 33113351

[ref25] Gerritsen J. Hornung B. Renckens B. van Hijum S. A. F. T. Martins Dos Santos V. A. P. Rijkers G. T. . (2017). Genomic and functional analysis of *Romboutsia ilealis* CRIB Treveals adaptation to the small intestine. PeerJ 5:e3698. doi: 10.7717/peerj.3698, 28924494 PMC5598433

[ref26] Ghosh T. S. Shanahan F. O’Toole P. W. (2022). The gut microbiome as a modulator of healthy ageing. Nat. Rev. Gastroenterol. Hepatol. 19, 565–584. doi: 10.1038/s41575-022-00605-x, 35468952 PMC9035980

[ref27] Goodrich J. K. Davenport E. R. Beaumont M. Jackson M. A. Knight R. Ober C. . (2016). Genetic determinants of the gut microbiome in UK twins. Cell Host Microbe 19, 731–743. doi: 10.1016/j.chom.2016.04.017, 27173935 PMC4915943

[ref28] Gouveia M. I. M. D. Bernalier-Donadille A. Jubelin G. (2024). Enterobacteriaceae in the human gut: dynamics and ecological roles in health and disease. Biology 13:142. doi: 10.3390/biology13030142 38534413 PMC10967970

[ref29] Guo S. Al-Sadi R. Said H. M. Ma T. Y. (2013). Lipopolysaccharide causes an increase in intestinal tight junction permeability in vitro and in vivo by inducing enterocyte membrane expression and localization of TLR-4 and CD14. Am. J. Pathol. 182, 375–387. doi: 10.1016/j.ajpath.2012.10.014, 23201091 PMC3562736

[ref30] Guo L. Wu Y. Wang C. Wei H. Tan J. Sun H. . (2020). Gut microbiological disorders reduce semen utilization rate in duroc boars. Front. Microbiol. 11:581926. doi: 10.3389/fmicb.2020.581926, 33133051 PMC7578402

[ref31] Han H. Zhou Y. Xiong B. Zhong R. Jiang Y. Sun H. . (2022). Alginate oligosaccharides increase boar semen quality by affecting gut microbiota and metabolites in blood and sperm. Front. Microbiol. 13:982152. doi: 10.3389/fmicb.2022.982152, 36071975 PMC9441641

[ref32] Hu R. Yang X. Gong J. Lv J. Yuan X. Shi M. . (2024). Patterns of alteration in boar semen quality from 9 to 37 months old and improvement by protocatechuic acid. J Anim Sci Biotechnol 15:78. doi: 10.1186/s40104-024-01031-6, 38755656 PMC11100174

[ref33] Huang L. Chen C. (2023). Employing pigs to decipher the host genetic effect on gut microbiome: advantages, challenges, and perspectives. Gut Microbes 15:2205410. doi: 10.1080/19490976.2023.2205410, 37122143 PMC10153013

[ref34] Kaltsas A. Giannakodimos I. Markou E. Stavropoulos M. Deligiannis D. Kratiras Z. . (2025). The Androbactome and the gut microbiota-testis Axis: a narrative review of emerging insights into male fertility. Int. J. Mol. Sci. 26:6211. doi: 10.3390/ijms26136211, 40649988 PMC12249747

[ref35] Kiššová Z. Tkáčiková Ľ. Mudroňová D. Bhide M. R. (2022). Immunomodulatory effect of *Lactobacillus reuteri* (Limosilactobacillus reuteri) and its exopolysaccharides investigated on epithelial cell line IPEC-J2 challenged with *Salmonella Typhimurium*. Life 12:1955. doi: 10.3390/life12121955, 36556320 PMC9788328

[ref36] Kurilshikov A. Medina-Gomez C. Bacigalupe R. Radjabzadeh D. Wang J. Demirkan A. . (2021). Large-scale association analyses identify host factors influencing human gut microbiome composition. Nat. Genet. 53, 156–165. doi: 10.1038/s41588-020-00763-1, 33462485 PMC8515199

[ref37] Lebas M. Garault P. Carrillo D. Codoñer F. M. Derrien M. (2020). Metabolic response of *Faecalibacterium prausnitzii* to cell-free supernatants from lactic acid Bacteria. Microorganisms 8:1528. doi: 10.3390/microorganisms8101528, 33027936 PMC7650636

[ref38] Lei T. Yang Y. Yang W.-X. (2025). Luteinizing hormone regulates testosterone production, Leydig cell proliferation, differentiation, and circadian rhythm during spermatogenesis. Int. J. Mol. Sci. 26:3548. doi: 10.3390/ijms26083548, 40332028 PMC12027374

[ref39] Lenoir M. Martín R. Torres-Maravilla E. Chadi S. González-Dávila P. Sokol H. . (2020). Butyrate mediates anti-inflammatory effects of *Faecalibacterium prausnitzii* in intestinal epithelial cells through Dact3. Gut Microbes 12, 1826748–1826716. doi: 10.1080/19490976.2020.1826748, 33054518 PMC7567499

[ref40] Li H. Durbin R. (2009). Fast and accurate short read alignment with burrows-wheeler transform. Bioinformatics 25, 1754–1760. doi: 10.1093/bioinformatics/btp324, 19451168 PMC2705234

[ref41] Lin Y. Wang K. Che L. Fang Z. Xu S. Feng B. . (2022). The improvement of semen quality by dietary Fiber intake is positively related with gut microbiota and SCFA in a boar model. Front. Microbiol. 13:863315. doi: 10.3389/fmicb.2022.863315, 35633720 PMC9130837

[ref42] Liufu S. Wang K. Chen B. Chen W. Liu X. Wen S. . (2024). Effect of host breeds on gut microbiome and fecal metabolome in commercial pigs. BMC Vet. Res. 20:458. doi: 10.1186/s12917-024-04308-0, 39390513 PMC11465751

[ref43] Lopera-Maya E. A. Kurilshikov A. Graaf A. V. D. Hu S. Andreu-Sánchez S. Chen L. . (2022). Effect of host genetics on the gut microbiome in 7,738 participants of the Dutch microbiome project. Nat. Genet. 54, 143–151. doi: 10.1038/s41588-021-00992-y, 35115690

[ref44] Lopez-Siles M. Duncan S. H. Garcia-Gil L. J. Martinez-Medina M. (2017). *Faecalibacterium prausnitzii*: from microbiology to diagnostics and prognostics. ISME J. 11, 841–852. doi: 10.1038/ismej.2016.176, 28045459 PMC5364359

[ref45] Lou Y. Lv Y. Wang X. Luo Y. Lou J. Yu Y. . (2025). *Ruminococcus torques* ameliorates the inflammation bowel disease and gut barrier dysfunction by modulating gut microbiota and bile acid metabolism. J. Transl. Med. 23:1162. doi: 10.1186/s12967-025-07192-w, 41131583 PMC12548225

[ref46] Lu X.-M. Lu P.-Z. Zhang H. (2013). Bacterial communities in manures of piglets and adult pigs bred with different feeds revealed by 16S rDNA 454 pyrosequencing. Appl. Microbiol. Biotechnol. 98, 2657–2665. doi: 10.1007/s00253-013-5211-4, 24068333

[ref47] Luo Y. Lan C. Li H. Ouyang Q. Kong F. Wu A. . (2022). Rational consideration of *Akkermansia muciniphila* targeting intestinal health: advantages and challenges. npj Biofilm. Microb. 8, 81–11. doi: 10.1038/s41522-022-00338-4, 36253412 PMC9576740

[ref48] Ma J. Chen J. Gan M. Chen L. Zhao Y. Zhu Y. . (2022). Gut microbiota composition and diversity in different commercial swine breeds in early and finishing growth stages. Animals 12:1607. doi: 10.3390/ani12131607, 35804507 PMC9264831

[ref49] O’Toole P. W. Jeffery I. B. (2015). Gut microbiota and aging. Science 350, 1214–1215. doi: 10.1126/science.aac8469 26785481

[ref50] O'Toole P. W. Claesson M. J. (2010). Gut microbiota: changes throughout the lifespan from infancy to elderly. Int. Dairy J. 20, 281–291. doi: 10.1016/j.idairyj.2009.11.010

[ref51] Ramaswamy S. Weinbauer G. F. (2014). Endocrine control of spermatogenesis: role of FSH and LH/ testosterone. Spermatogenesis 4:e996025. doi: 10.1080/21565562.2014.996025, 26413400 PMC4581062

[ref52] Rothschild D. Weissbrod O. Barkan E. Kurilshikov A. Korem T. Zeevi D. . (2018). Environment dominates over host genetics in shaping human gut microbiota. Nature 555, 210–215. doi: 10.1038/nature25973, 29489753

[ref53] Rowland I. Gibson G. Heinken A. Scott K. Swann J. Thiele I. . (2017). Gut microbiota functions: metabolism of nutrients and other food components. Eur. J. Nutr. 57, 1–24. doi: 10.1007/s00394-017-1445-8, 28393285 PMC5847071

[ref54] Sá P. Godinho R. M. Gòdia M. Sevillano C. A. Harlizius B. Madsen O. . (2025). The effect of aging on genetic parameters of boar semen traits. J. Anim. Sci. 103:skaf257. doi: 10.1093/jas/skaf257, 40747796 PMC12445636

[ref55] Sanna S. Kurilshikov A. van der Graaf A. Fu J. Zhernakova A. (2022). Challenges and future directions for studying effects of host genetics on the gut microbiome. Nat. Genet. 54, 100–106. doi: 10.1038/s41588-021-00983-z, 35115688

[ref56] Sayavedra L. Yasir M. Goldson A. Brion A. Gall G. L. Moreno-Gonzalez M. . (2025). Bacterial microcompartments and energy metabolism drive gut colonization by *Bilophila wadsworthia*. Nat. Commun. 16:5049. doi: 10.1038/s41467-025-60180-y, 40447622 PMC12125255

[ref57] Sciellour M. L. Renaudeau D. Zemb O. (2019). Longitudinal analysis of the microbiota composition and enterotypes of pigs from post-weaning to finishing. Microorganisms 7:622. doi: 10.3390/microorganisms7120622, 31795103 PMC6956163

[ref58] Sekirov I. Russell S. L. Antunes L. C. M. Finlay B. B. (2010). Gut microbiota in health and disease. Physiol. Rev. 90, 859–904. doi: 10.1152/physrev.00045.2009 20664075

[ref59] Shkoporov A. N. Chaplin A. V. Shcherbakova V. A. Suzina N. E. Kafarskaia L. I. Bozhenko V. K. . (2016). Ruthenibacterium lactatiformans gen. Nov., sp. nov., an anaerobic, lactate-producing member of the family Ruminococcaceae isolated from human faeces. Int. J. Syst. Evol. Microbiol. 66, 3041–3049. doi: 10.1099/ijsem.0.001143, 27154556

[ref60] Spaziani M. Tarantino C. Tahani N. Gianfrilli D. Sbardella E. Lenzi A. . (2020). Hypothalamo-pituitary axis and puberty. Mol. Cell. Endocrinol. 520:111094. doi: 10.1016/j.mce.2020.111094, 33271219

[ref61] Stewart C. J. Ajami N. J. O’Brien J. L. Hutchinson D. S. Smith D. P. Wong M. C. . (2018). Temporal development of the gut microbiome in early childhood from the TEDDY study. Nature 562, 583–588. doi: 10.1038/s41586-018-0617-x, 30356187 PMC6415775

[ref62] Trachsel J. Humphrey S. Allen H. K. (2018). Butyricicoccus porcorum sp. nov., a butyrate-producing bacterium from swine intestinal tract. Int. J. Syst. Evol. Microbiol. 68, 1737–1742. doi: 10.1099/ijsem.0.002738, 29620502

[ref63] Tsakmakidis I. A. Khalifa T. A. Boscos C. M. (2012). Age-related changes in quality and fertility of porcine semen. Biol. Res. 45, 381–386. doi: 10.4067/s0716-97602012000400009, 23558995

[ref64] Turpin W. Espin-Garcia O. Xu W. Silverberg M. S. Kevans D. Smith M. I. . (2016). Association of host genome with intestinal microbial composition in a large healthy cohort. Nat. Genet. 48, 1413–1417. doi: 10.1038/ng.3693 27694960

[ref65] Venegas D. P. Fuente M. K. D. L. Landskron G. González M. J. Quera R. Dijkstra G. . (2019). Short Chain fatty acids (SCFAs)-mediated gut epithelial and immune regulation and its relevance for inflammatory bowel diseases. Front. Immunol. 10:277. doi: 10.3389/fimmu.2019.00277, 30915065 PMC6421268

[ref66] Vishnu Prasoodanan P. K. Sharma A. K. Mahajan S. Dhakan D. B. Maji A. Scaria J. . (2021). Western and non-western gut microbiomes reveal new roles of Prevotella in carbohydrate metabolism and mouth–gut axis. npj Biofil. Microb. 7, 77–17. doi: 10.1038/s41522-021-00248-x, 34620880 PMC8497558

[ref67] Walke G. Gaurkar S. S. Prasad R. Lohakare T. Wanjari M. (2023). The impact of oxidative stress on male reproductive function: exploring the role of antioxidant supplementation. Cureus 15:e42583. doi: 10.7759/cureus.42583, 37641770 PMC10460465

[ref68] Wang X. Tsai T. Deng F. Wei X. Chai J. Knapp J. . (2019). Longitudinal investigation of the swine gut microbiome from birth to market reveals stage and growth performance associated bacteria. Microbiome 7, 109–118. doi: 10.1186/s40168-019-0721-7, 31362781 PMC6664762

[ref69] Wu T. Hu E. Xu S. Chen M. Guo P. Dai Z. . (2021). clusterProfiler 4.0: a universal enrichment tool for interpreting omics data. The. Innovation 2:100141. doi: 10.1016/j.xinn.2021.100141, 34557778 PMC8454663

[ref70] Yang L. Bian G. Su Y. Zhu W. (2014). Comparison of faecal microbial Community of Lantang, Bama, Erhualian, Meishan, Xiaomeishan, duroc, landrace, and Yorkshire sows. Asian Australas. J. Anim. Sci. 27, 898–906. doi: 10.5713/ajas.2013.13621, 25050029 PMC4093183

[ref71] Yang H. Wu J. Huang X. Zhou Y. Zhang Y. Liu M. . (2022). ABO genotype alters the gut microbiota by regulating GalNAc levels in pigs. Nature 606, 358–367. doi: 10.1038/s41586-022-04769-z, 35477154 PMC9157047

[ref72] Yatsunenko T. Rey F. E. Manary M. J. Trehan I. Dominguez-Bello M. G. Contreras M. . (2012). Human gut microbiome viewed across age and geography. Nature 486, 222–227. doi: 10.1038/nature11053, 22699611 PMC3376388

[ref73] Youngblut N. D. Reischer G. H. Walters W. Schuster N. Walzer C. Stalder G. . (2018). Host diet and evolutionary history explain different aspects of gut microbiome diversity among vertebrate clades. Nat. Commun. 10:2200. doi: 10.1038/s41467-019-10191-3, 31097702 PMC6522487

[ref74] Yu J. Zhao P. Zheng X. Zhou L. Wang C. Liu J.-F. (2020). Genome-wide detection of selection signatures in duroc revealed candidate genes relating to growth and meat quality. G3 Bethesda 10, 3765–3773. doi: 10.1534/g3.120.401628, 32859686 PMC7534417

[ref75] Zhang J. Guo Z. Xue Z. Sun Z. Zhang M. Wang L. . (2015). A phylo-functional core of gut microbiota in healthy young Chinese cohorts across lifestyles, geography and ethnicities. ISME J. 9, 1979–1990. doi: 10.1038/ismej.2015.11, 25647347 PMC4542028

[ref76] Zhang H. Wielen N. V. D. Hee B. V. D. Wang J. Hendriks W. Gilbert M. (2020). Impact of fermentable protein, by feeding high protein diets, on microbial composition, microbial catabolic activity, gut health and beyond in pigs. Microorganisms 8:1735. doi: 10.3390/microorganisms8111735, 33167470 PMC7694525

[ref77] Zhao S. Lau R. Zhong Y. Chen M.-H. (2024). Lactate cross-feeding between Bifidobacterium species and Megasphaera indica contributes to butyrate formation in the human colonic environment. Appl. Environ. Microbiol. 90:e0101923. doi: 10.1128/aem.01019-23, 38126785 PMC10807433

[ref78] Zhou Y. Chen L. Han H. Xiong B. Zhong R. Jiang Y. . (2022). Taxifolin increased semen quality of duroc boars by improving gut microbes and blood metabolites. Front. Microbiol. 13:1020628. doi: 10.3389/fmicb.2022.1020628, 36312933 PMC9614168

