# Supplementary figures and images for: Revealing gut microbiota profiles and their influencing factors in commercial boars of three breeds by a large-scale metagenome study
Source: Front Microbiol. 2026 May 20;17:1825304. doi: 10.3389/fmicb.2026.1825304 (PMC13230162; doi:10.3389/fmicb.2026.1825304)

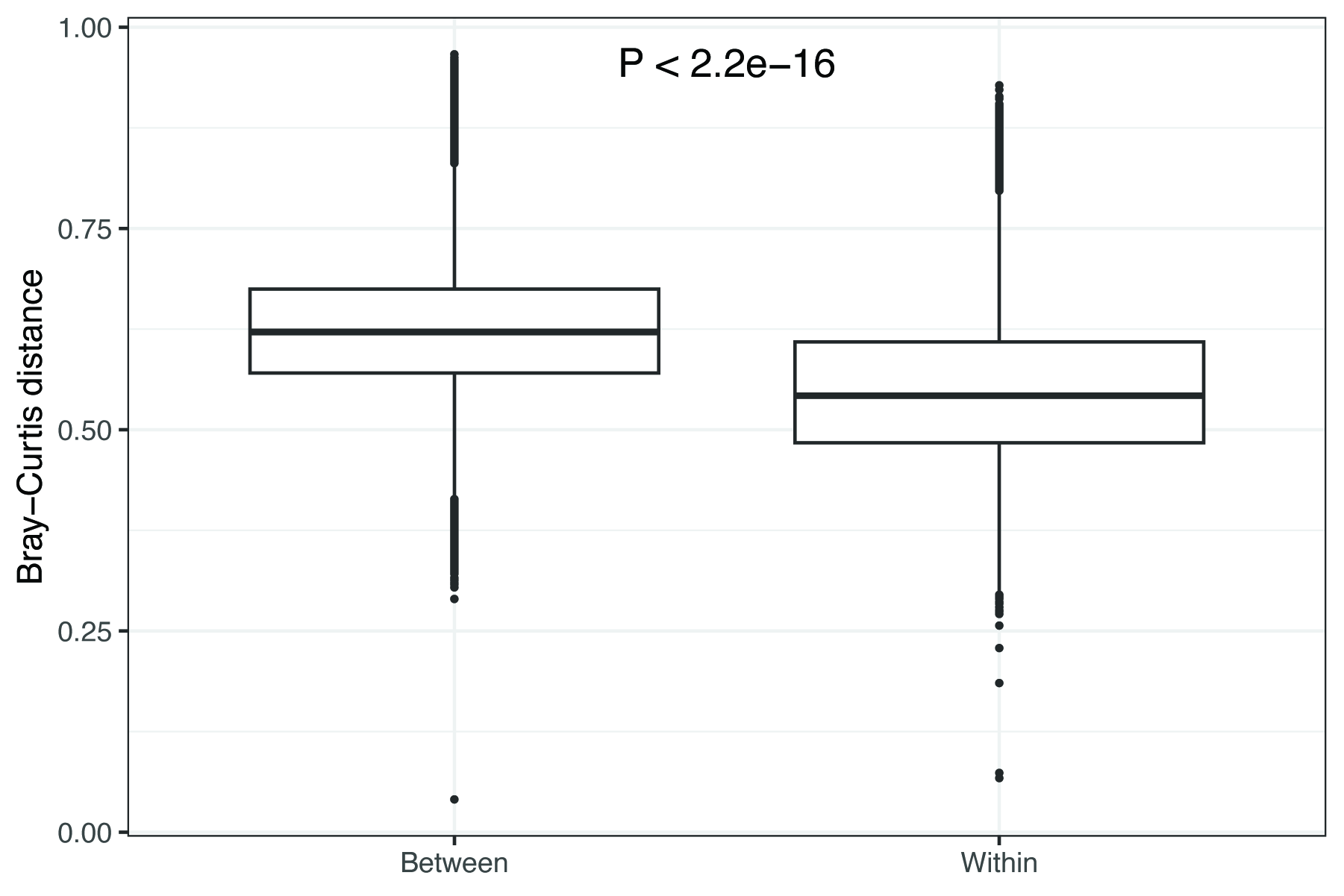

Supplement: SUPPLEMENTARY FIGURE 1 — Boxplot showing within-breed and between-breed differences based on Bray–Curtis distances corresponding to the PCoA in Figure 2D. Statistical significance is indicated by asterisks (*P < 0.05, **P < 0.01, and ***P < 0.001). [file Image_1.tif]

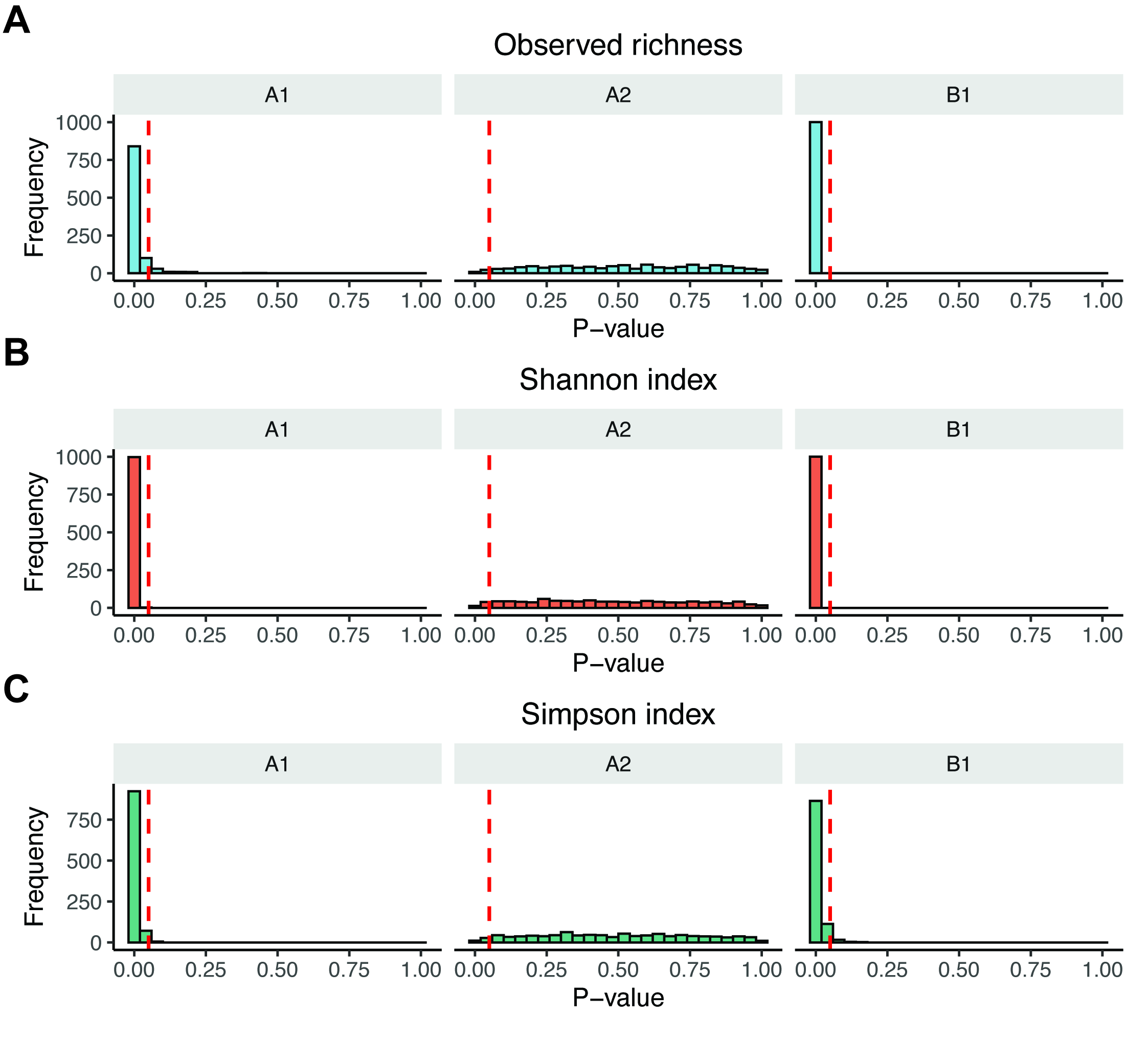

Supplement: SUPPLEMENTARY FIGURE 2 — Distribution of P-values from the comparison of the α-diversity among breeds across 1,000 bootstrap-based resampling iterations within each farm. To control for unequal sample sizes among breeds within farms, individuals from each breed were randomly subsampled to the size of the smallest breed group in the corresponding farm, and the procedure was repeated 1,000 times. Histograms show the distributions of P-values obtained from Kruskal–Wallis tests for Observed richness (A), Shannon index (B), and Simpson index (C) in A1, A2, and B1 farms. The red dashed line indicates the significance threshold at P = 0.05. [file Image_2.tif]

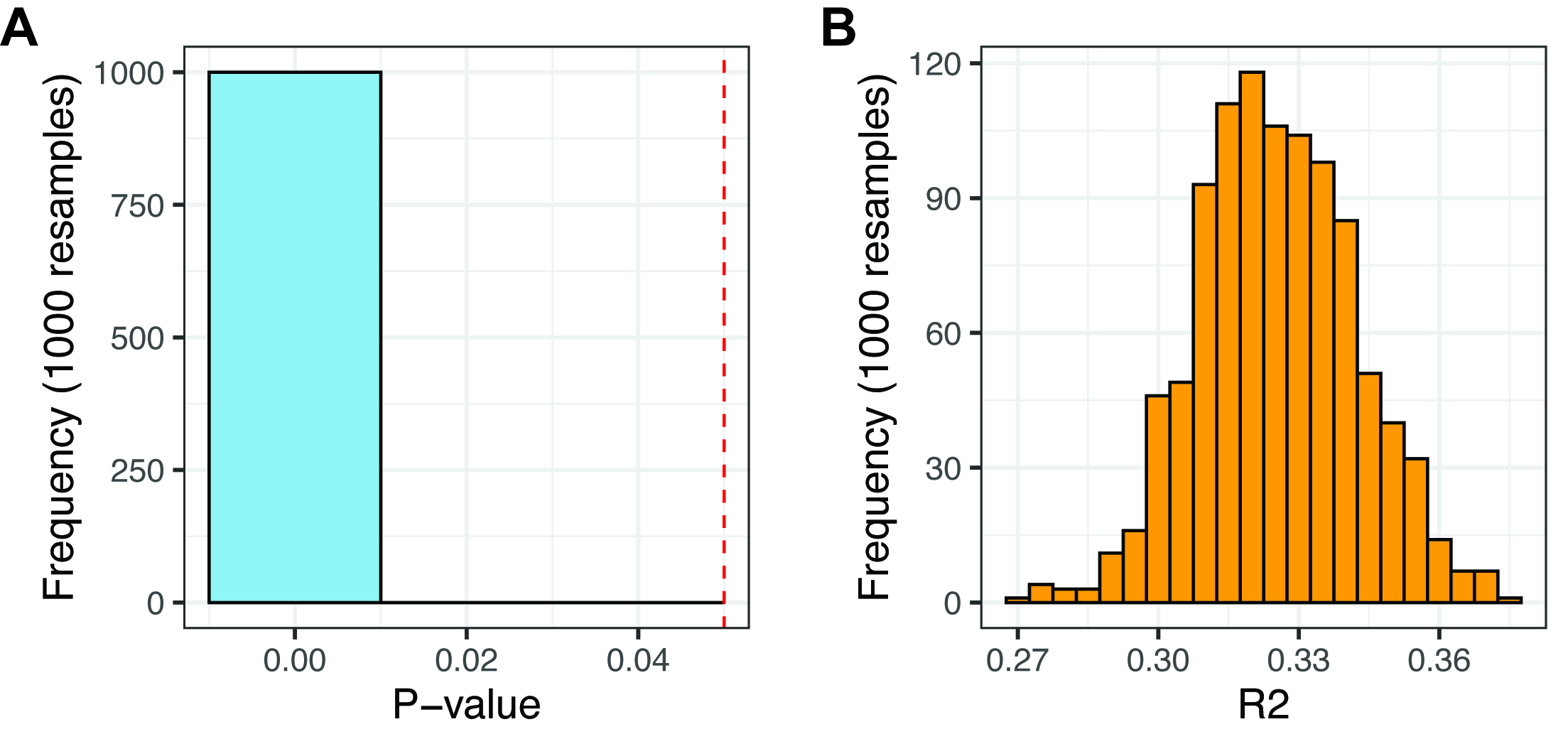

Supplement: SUPPLEMENTARY FIGURE 3 — Distribution of PERMANOVA results for β-diversity across 1,000 resampling iterations. Individuals in each breed from all three farms were randomly subsampled to a common sample size defined by the smallest group across all breeds and farms, and the analysis was repeated 1,000 times. (A) Histogram of P-values obtained from PERMANOVA across resampling iterations. The red dashed line indicates the significance threshold at P = 0.05. (B) Distribution of the corresponding R² values, representing the proportion of variance in microbial community structure explained by group differences. The consistent enrichment of significant P-values together with stable R² distributions indicates that the observed differences in community composition are robust to sample size normalization. [file Image_3.tif]

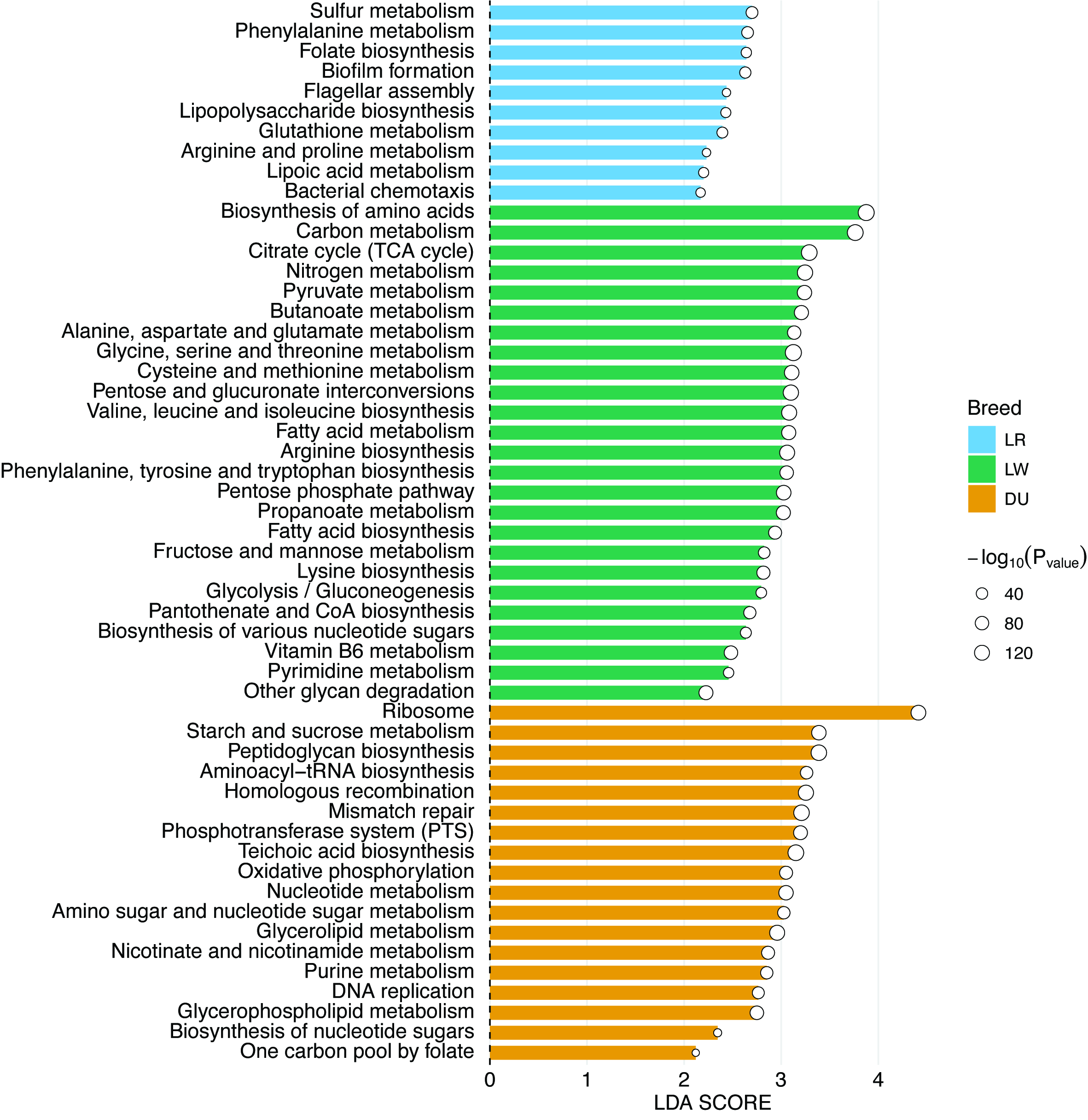

Supplement: SUPPLEMENTARY FIGURE 4 — Differentially enriched functional pathways of the gut microbiome among three pig breeds identified by LEfSe analysis. Pathway abundance profiles derived from HUMAnN annotation were used to identify differentially enriched metabolic pathways among Duroc, Landrace, and Large White boars. The x-axis indicates the LDA score, and the y-axis shows the pathway names. Bar colors represent the breed in which each pathway was enriched, with blue for Landrace, green for Large White, and orange for Duroc. The size of the open circles indicates the significance level of each pathway, expressed as -log10(P-value). Pathways within each breed are ordered from high to low according to the LDA score. [file Image_4.tif]

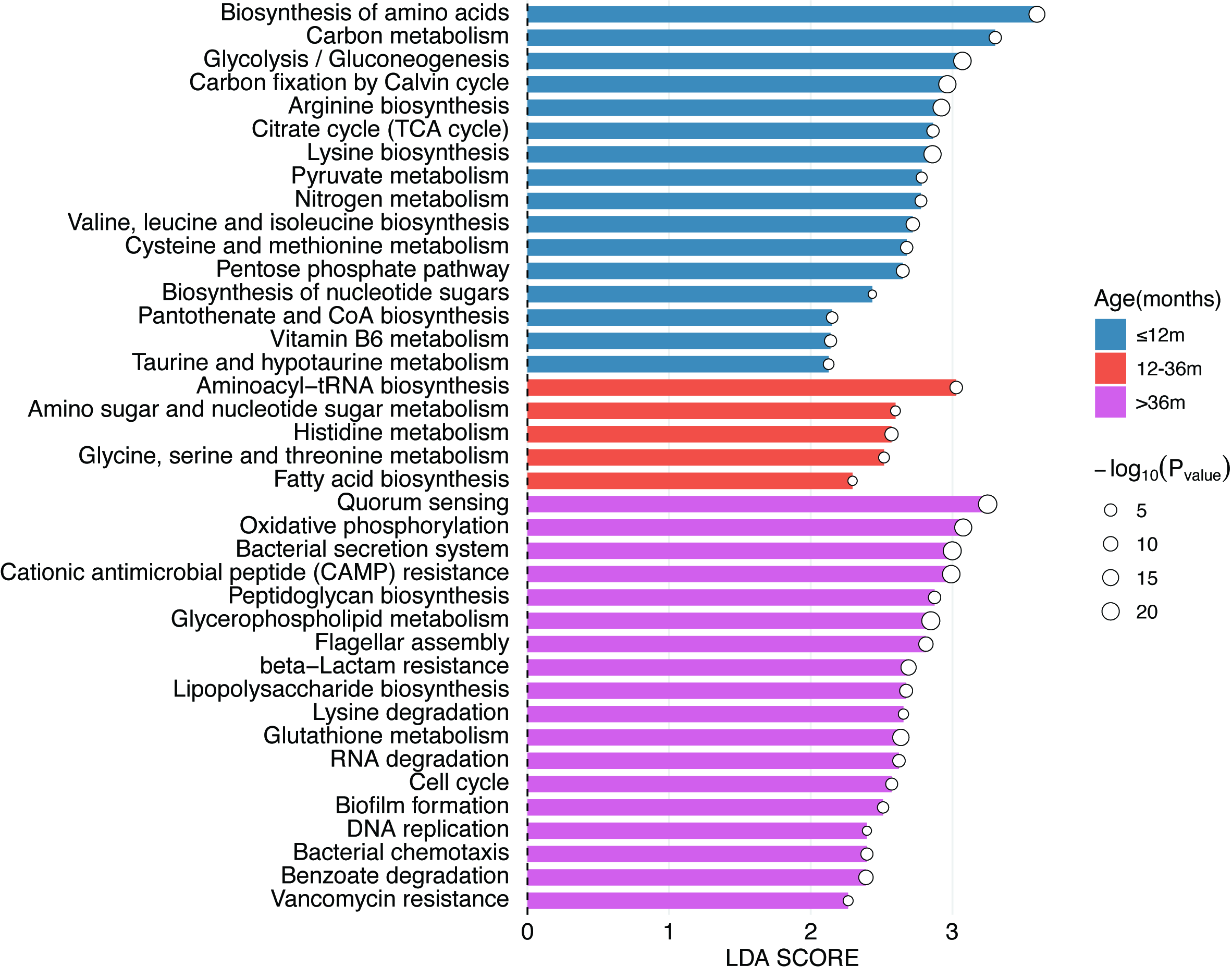

Supplement: SUPPLEMENTARY FIGURE 5 — Differentially enriched functional pathways of the gut microbiome across three age stages identified by LEfSe analysis. Pathway abundance profiles derived from HUMAnN annotation were used to identify differentially enriched metabolic pathways among young, mature, and aged boars. The x-axis indicates the LDA score, and the y-axis shows the pathway names. Bar colors represent the age stage in which each pathway was enriched, with blue for young boars, orange for mature boars, and pink for aged boars. The size of the open circles indicates the significance level of each pathway, expressed as -log10(P-value). Pathways within each age stage are ordered from high to low according to the LDA score. [file Image_5.tif]
